# Recent advances in biomass deconstruction, microbial conversion, artificial intelligence, and carbon capture for sustainable bioenergy

**DOI:** 10.1186/s40643-026-01081-w

**Published:** 2026-06-11

**Authors:** Vishwajit Kumar, Shikha Mishra, Pratham Joshi, Jyotsna Misra, Prakash Peralam Yegneswaran, Bhavanari Mallikarjun, Syed Shams Yazdani, Piyush Behari Lal

**Affiliations:** 1 Department of Microbiology, Kasturba Medical College, Manipal Academy of Higher Education, Manipal, Karnataka 576104 India; 2 Department of Biotechnology, Manipal Institute of Technology, Manipal Academy of Higher Education, Manipal, Karnataka 576104 India; 3 International Centre for Genetic Engineering and Biotechnology, New Delhi, 110067 India; 4 Department of Biotechnology, Graphic Era Deemed to Be University, Dehradun, Uttarakhand 248002 India; 5 Manipal Institute of Technology, Manipal Academy of Higher Education, Manipal, Karnataka 576104 India

**Keywords:** Lignocellulosic biomass, Pretreatment & Enzymatic deconstruction, Microbial conversion, Carbon capture, Consolidated bioprocessing, Artificial intelligence

## Abstract

The transition from fossil fuels to low-carbon bio-based energy systems is increasingly constrained by the efficiency, scalability, and integration of conversion technologies. Addressing this challenge, this review critically analyzes microbial biofuel production through a conversion-centric and systems-level framework, emphasizing how feedstock diversity, pretreatment chemistry, enzymatic deconstruction, and microbial metabolism collectively govern overall process performance. This review evaluates how pretreatment strategies modulate biomass recalcitrance, hydrolysate chemical ecologies, inhibitor profiles, and redox balance, thereby imposing fundamental constraints on enzymatic efficiency, microbial conversion yields, and emissions outcomes. Advances in enzymatic hydrolysis are assessed in terms of bond-specific catalysis, enzyme synergy, and persistent bottlenecks arising from substrate heterogeneity, lignin enzyme interactions, and non-productive binding, while microbial engineering strategies from robust monocultures to synthetic consortia and cell-free systems are examined through techno-economic and metabolic flux perspectives. It further highlights the emerging role of artificial intelligence and multi-omics integration in enabling predictive optimization of pretreatment severity, enzyme cocktails, and metabolic routing, moving beyond empirical process tuning. This article establishes a unified framework for integrated “microbial lignocellulose-to-fuel” pathways, demonstrating how coordinated advances in conversion technologies are essential for achieving scalable, economically viable, and environmentally sustainable bioenergy systems.

**Graphical abstract:**

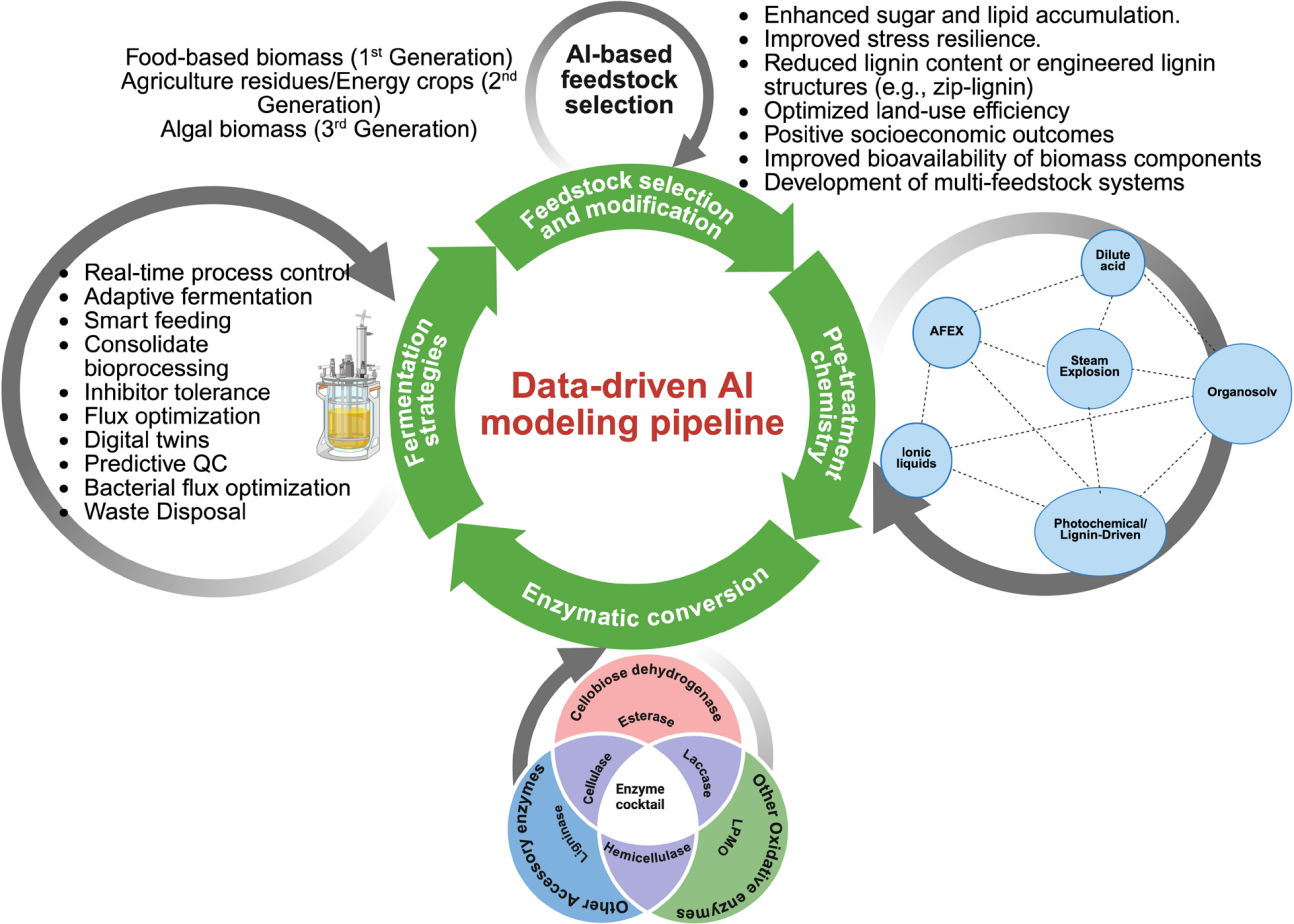

**Supplementary Information:**

The online version contains supplementary material available at 10.1186/s40643-026-01081-w.

## Introduction

Biofuel production from agricultural biomass is a complex, multi-step process involving feedstock selection and modification, pretreatment, enzymatic deconstruction, and microbial fermentation. Each stage is influenced by numerous physicochemical, biological, and process variables that must be carefully optimized to achieve efficient, scalable, and economically viable biofuel production (Samantaray et al. 2024). The expanding spectrum of biomass resources, from agricultural residues and forestry by-products to dedicated energy crops, agro-industrial wastes, microalgae, industrial effluents, and C1/C2 gases, adds significant complexity but also enables regional adaptability and greater supply-chain resilience (Sanoja-Lopez et al. 2024). Several articles have documented these diverse feedstocks that can be converted into biofuels through complementary biological and chemical pathways, offering renewable alternatives with reduced net carbon emissions and environmental impact (Lynd et al. 2008; Bedru et al. 2025; Abdul Hakim Shaah et al. 2021; Inyang et al. 2022). The inclusion of emerging feedstocks such as rice straw, sugar beet pulp, microalgae, and municipal solid waste expands opportunities for waste utilization and integrated biorefineries, stimulating innovation in pretreatment, bioconversion, and electro-biological coupling (Rumbold et al. 2010). However, many region-specific, low-cost biomass sources remain underexplored, representing untapped potential for next-generation biofuel systems (Fischer et al. 2008). Equally important is the selection of an appropriate pretreatment strategy and microbial system, as it directly determines conversion efficiency and final fuel yield. The biochemical makeup of the feedstock, especially its carbohydrate, lignin, and lipid content, strongly shapes both microbial pathway choice and processing strategy. High-carbohydrate substrates readily support fermentative production of fuels such as ethanol or butanol, whereas more complex or low-carbohydrate materials require specialized microorganisms and customized metabolic engineering approaches. A detailed understanding of these compositional features is therefore vital for choosing or designing effective biocatalysts for optimal biofuel production.

As experimental data across feedstocks, pretreatment strategies, enzyme systems, and microbial platforms continue to grow, artificial intelligence (AI) and machine learning (ML) have become essential for integrating this complexity and guiding rational process design. AI-driven approaches enable predictive feedstock characterization, pretreatment optimization, and multi-omics-guided strain and enzyme engineering, while also supporting the design of synthetic consortia and advanced fermentation strategies (Liao and Yao 2021a; Okolie 2024; Nair and Verma 2025). At the systems level, AI-assisted techno-economic (TEA) and life-cycle analyses (LCA) inform scalability, cost efficiency, and environmental sustainability, accelerating the development of integrated and circular bioenergy technologies (Fu et al. 2023). This review highlights key parameters across the biofuel production pipeline, including feedstock diversity, pretreatment innovations, enzymatic and microbial conversion strategies, and AI-guided process design. A comprehensive understanding of these interconnected variables is essential for building robust, scalable, and commercially viable bioenergy systems that are both economically and environmentally sustainable.

## From first-generation fuels to multi-feedstock platforms

The evolution of biofuel production has progressed from food-based first-generation systems to engineered, multi-feedstock, fourth-generation platforms optimized with synthetic biology and microbial engineering. This trajectory reflects a continuous effort to balance productivity, sustainability, and ecological integrity. First-generation biofuels depended largely on food-based, high-sugar or starch crops such as corn, sugarcane, and vegetable oils. Although these substrates supported efficient fermentation, their use raised significant ethical and environmental concerns related to food-fuel competition and intensive resource consumption (S N Naik et al. 2010). In response, second-generation biofuels utilized lignocellulosic biomass such as agricultural residues, forestry by-products, and energy crops, thus decoupling energy production from food supply but introducing new technical challenges in biomass recalcitrance, lignin removal, and enzymatic hydrolysis efficiency (Kammoun et al. 2023). Third-generation algae-based systems provided high productivity and CO₂ recycling potential, while fourth-generation biofuels integrate synthetic biology, genetic engineering, and carbon capture to create climate-positive, high-yield systems. Yet, across all generations, the type and treatment of feedstock dictate the biology required downstream processing (Chowdhury and Loganathan 2019).

Among these advances, hybrid feedstock systems, that combines multiple feedstocks, are emerging as a pivotal innovation for ensuring continuous, economically viable, and sustainable biofuel production (Sharma and Singh 2010). By combining diverse biomass streams, hybrid systems balance compositional heterogeneity, stabilize seasonal supply, and improve conversion efficiency. Co-blending lignocellulosic materials with carbohydrate or lipid-rich substrates enhances fermentable sugar yields, moderates pretreatment severity, and enables flexible product targeting. For example, integrating sugarcane bagasse with food waste or microalgae supplies, both carbon-rich and hydrogen-dense fractions, improves bioethanol and biodiesel synthesis simultaneously (Begum et al. 2024). These approaches reduce dependence on monocultures, mitigate land-use conflicts, and embody circular bioeconomy principles by utilizing waste feedstocks that would otherwise contribute to pollution (Diakosavvas and Frezal 2019). Integrating hybrid feedstock strategies with AI and ML-driven pretreatment–biology frameworks enables the coordinated design of biomass processing and microbial engineering for advanced biofuels. ML-guided feedstock blending, combined with downstream processes, enhances resource utilization while maintaining biodiversity and socioeconomic balance, reducing reliance on trial-and-error approaches (Ahmad et al. 2021; Smuga-Kogut et al. 2021). Together, these strategies create predictive, modular, and resilient feedstock selection systems, where feedstock composition, biochemical properties, bioavailability, and socioeconomic considerations directly inform the efficient and sustainable use of biomass resources.

### Commonly used feedstocks

Advantages and limitations of plant-based feedstocks have been well documented (Vohra et al. 2014; Bušić et al. 2018; Jain and Kumar 2024) (Table S1). However, integrating multiple feedstocks into bioenergy pipelines requires careful consideration of factors such as spatial and seasonal availability, intrinsic properties like carbohydrates and lignin content, and overall suitability for bioconversion. Diversity in bioenergy species enables continuous biorefinery feedstock supply and mitigates land-use pressure and seasonal constraints. However, such heterogeneity generates high-dimensional compositional and structural datasets that must be integrated into AI-driven decision frameworks to guide pretreatment design, enzyme selection, and microbial processing. Food-based biomass, agricultural residues, and non-food energy crops collectively constitute a scalable feedstock portfolio whose efficient utilization requires data-driven, feedstock-specific biorefinery engineering for predictable, high-yield biofuel conversion (Selwal et al. 2025).

### Food-based biomass as feedstock

Food-based biomass remains central to global biofuel deployment due to its high carbohydrate or lipid accessibility, established agronomic systems, and compatibility with mature conversion technologies. Among these, sugarcane, maize, and wheat dominate first-generation biofuel production, while selected oilseed crops contribute to biodiesel and renewable aviation fuels. However, the technical feasibility and sustainability of these feedstocks are governed by the alignment between biomass composition, conversion pathway, and resource intensity rather than availability alone.

Sugarcane-based systems are the most efficient platforms for bioethanol production, as sucrose-rich juice can be directly fermented with minimal treatment. Cultivated across ~ 26 million hectares in tropical and subtropical regions, sugarcane-based systems routinely achieve ethanol yields of 6,000–7,000 L ha⁻^1^ yr⁻^1^ at competitive production costs. High water demand and land-use competition further challenge the long-term sustainability of sugarcane-based biofuels (Tana et al. 2016; Bušić et al. 2018; Mahmud and Anannya 2021). Maize is another cornerstone of first-generation ethanol production, particularly in North America. The grain’s high starch content (68–72%) and low lignin facilitate efficient enzymatic saccharification and fermentation. While maize ethanol benefits from strong policy support and technological maturity, it raises concerns related to food security, fertilizer-intensive cultivation, and greenhouse gas emissions(Kim and Dale 2005; Kurambhatti et al. 2018; Amer et al. 2021). Wheat also plays a more regionally constrained role in biofuel production with similar constraints of “food vs fuel”. Its high starch content provides easy conversion into biofuel, but it competes directly with food supply chains (Saunders et al. 2011; Cripwell et al. 2015; Li et al. 2019). Contrast, non-food oilseed crops such as Camelina, Crambe, and Carinata are increasingly aligned with biodiesel and renewable aviation fuel production. Their high lipid content and favourable fatty acid profiles enable efficient thermochemical upgrading, with lower food-system competition and improved greenhouse gas mitigation potential (Shonnard et al. In^2010^; Chhikara et al. 2018).

Collectively, these crops provide a diverse and abundant feedstock biomass that may be utilized as a strategic asset for resilient and scalable biofuel systems (Sh Chhikara et al. 2018; Limbalkar et al. 2021). Overall, food-based biomass provides reliable and technologically mature routes for biofuel production, but its long-term sustainability is constrained by resource intensity and pathway specificity. Future bioenergy systems must therefore integrate food-based feedstocks within diversified, flexible biorefineries that balance conversion efficiency with environmental and socio-economic considerations.

### Agricultural residues

**Fig. 1:**
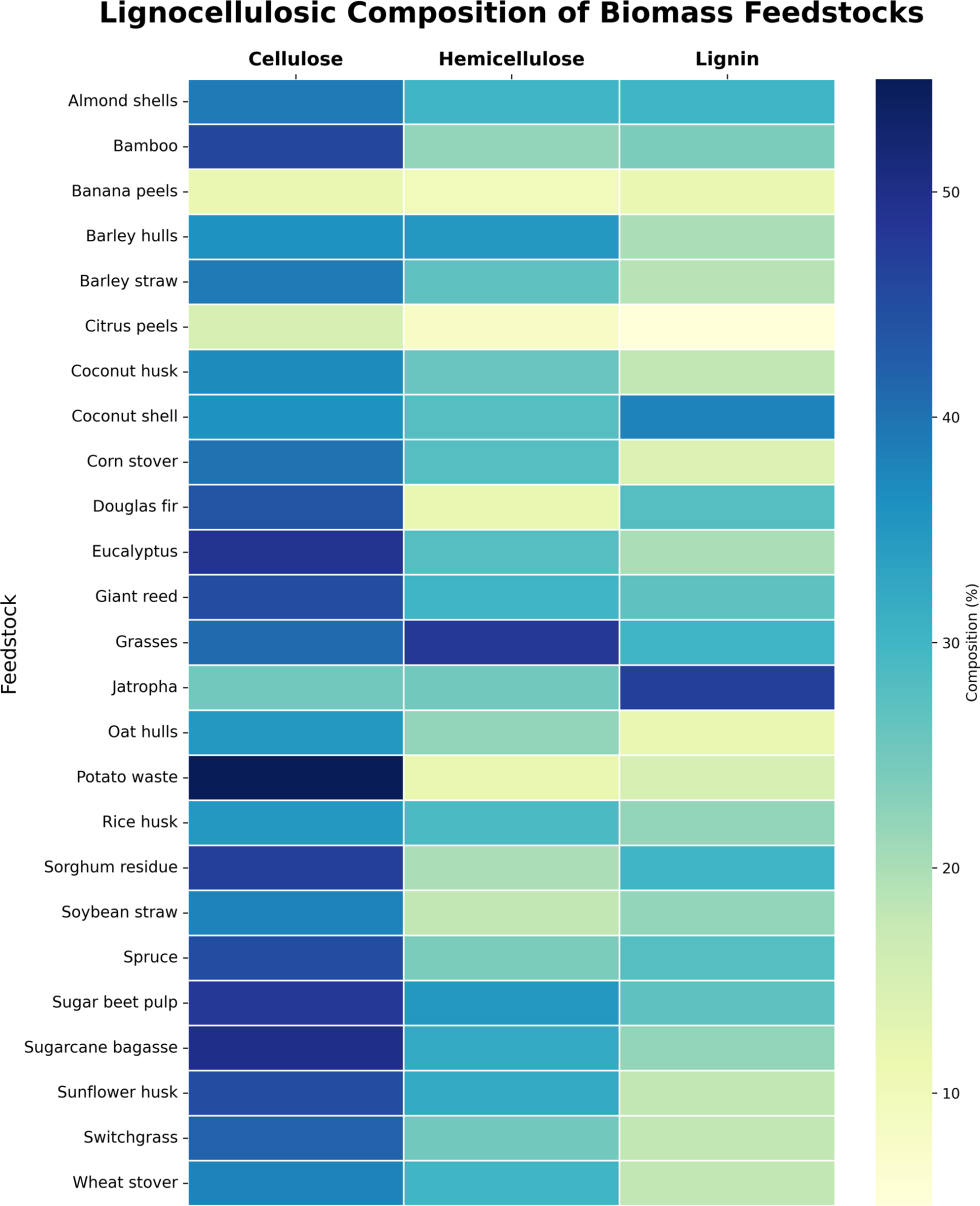
Heatmap illustrating the compositional variability of lignocellulosic biomass across diverse feedstocks, showing the relative abundance of cellulose, hemicellulose, and lignin. Component percentages were compiled from published literature (Table S2). The heatmap was generated using Python (Matplotlib v3.x) with color gradients representing relative abundance (%), highlighting feedstock-specific differences relevant to biomass pretreatment and conversion processes**.**

**Fig. 2:**
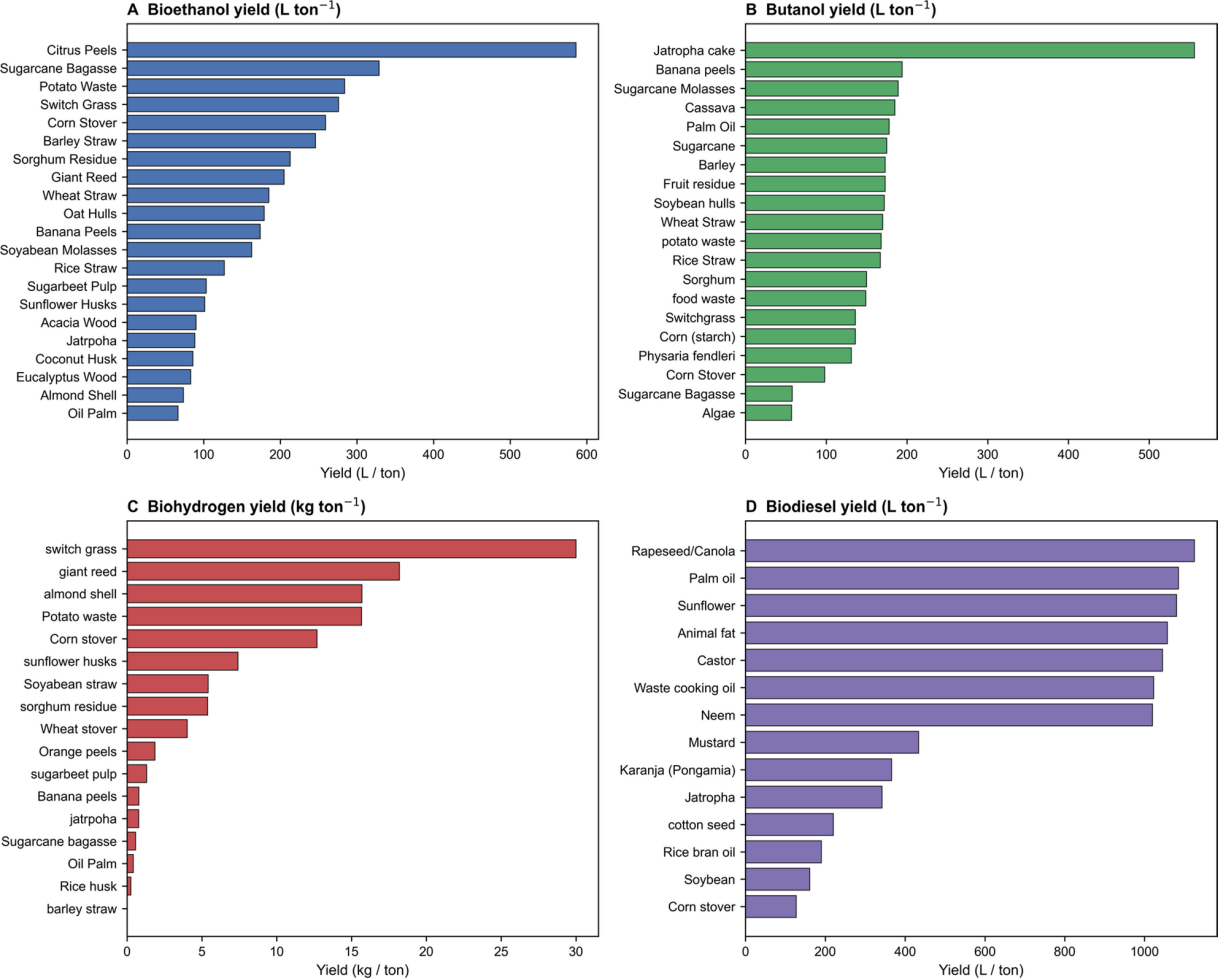
Comparative biofuel yields from diverse feedstocks. Bar graphs show the maximum reported yields of **a** bioethanol, **b** biobutanol, **c** biohydrogen, and **d** biodiesel. Yield data were compiled from published literature (Tables S3–S6). All values are normalized to a feedstock mass basis and were calculated by converting reported concentrations or mass yields using standard density and stoichiometric conversion factors. Figures were generated using Python (Matplotlib)

Agricultural residues constitute a vast and renewable source of lignocellulosic biomass with significant potential to diversify and scale sustainable biofuel production. A few representative examples are highlighted in Fig. 1, and supplementary Table S1. Major residues, such as corn stover, wheat straw, rice straw and husk, sugarcane bagasse, sorghum biomass, and coconut husk, are generated in large quantities across diverse agroclimatic regions and share comparable structural carbohydrate contents (typically 60–75% cellulose and hemicellulose) (Panthapulakkal and Sain 2015; Adhikari et al. 2018) (Supplementary Table S2). While some residues are already standardized for biofuel synthesis, many remain underutilized as standalone resources. Their use as complementary feedstocks alongside food-based substrates expands the bioenergy resource base, enhances feedstock and process flexibility, and supports the development of resilient, region-specific biorefineries. This feedstock diversity ensures year-round availability and reduces supply-chain risks associated with seasonality, climate variability, and competition with food or feed uses, thereby enabling the production of bioethanol, biobutanol, biomethane, biohydrogen, and other advanced biofuels (Fig. 2) (Badgujar and Bhanage 2018; Dhanraj et al. 2021).

From a technological perspective, variation in chemical composition, particularly in carbohydrate, lignin, ash, and silica content, requires alignment of specific feedstocks with appropriate pretreatment and conversion pathways, including biochemical, thermochemical, and hybrid processes. For example, corn stover is constrained by moderate lignin content and compositional heterogeneity, requiring careful pretreatment control, while wheat straw is limited by recalcitrant lignin carbohydrate complexes and seasonal availability. Rice straw and husk pose challenges due to high ash and silica levels, which reduce enzymatic hydrolysis efficiency and cause slagging and catalyst deactivation in thermochemical processes (Binod et al. 2010; Belal 2013; Hafez et al. 2023; Ningthoujam et al. 2023). Sugarcane bagasse, despite high carbohydrate content (Niju and Swathika 2019), is hindered by high moisture, compositional variability, and competition with cogeneration, limiting its use for advanced biofuels (Ojo-kupoluyi et al. 2024). Sorghum biomass offers high yields and soluble sugars but suffers from inefficient utilization of its lignocellulosic fraction (Mullet et al. 2014; Stamenković et al. 2023), whereas coconut husk is highly lignified (Van Dam et al. 2004), requiring intensive delignification and high energy input with suboptimal syngas quality (Bolivar-Telleria et al. 2018).

Variations in lignin content, ash and silica levels, and cell wall architecture strongly influence process efficiency across biochemical and thermochemical pathways. In biochemical routes, pretreatment is the major technical and economic bottleneck (Yang and Charles E Wyman 2008; Karimi et al. 2013; Satari et al. 2018; Galbe and Wallberg 2019). Conventional physicochemical pretreatments improve carbohydrate accessibility but generate inhibitory compounds such as furans, organic acids, and phenolics, reducing microbial productivity and increasing detoxification requirements (Fan et al. 2025). Feedstocks with high ash and silica contents, particularly rice straw and husk, pose additional challenges by limiting enzymatic hydrolysis and causing fouling and inefficiencies in thermochemical systems. Similarly, highly lignified materials such as coconut husk and moisture-rich substrates like sugarcane bagasse highlight the limitations of current one-size-fits-all conversion approaches (Khaleghian et al. 2017). Improving biofuel yields therefore requires low-severity, selective pretreatment strategies coupled with robust microbial platforms and integrated biorefinery designs to maximize carbon and energy recovery. Blending or spiking lignocellulosic residues with food-based biomass can further reduce pretreatment severity and enhance downstream process stability.

### Non-food energy crops

These specialized crops are cultivated specifically for energy generation rather than consumption. A few notable examples are highlighted in supplementary tables S3–S6. Non-food energy crops represent a strategic feedstock class for sustainable biofuel synthesis, as they provide substantial biomass without direct competition with food systems or land-use policies. Prominent examples are switchgrass, jatropha, bast fiber crops (jute and kenaf), miscanthus, short-rotation woody crops (SRWCs), bamboo, and algae. Technical analyses indicate that biofuel viability from these crops is governed less by nominal biomass yield or composition alone, and more by compatibility with specific conversion pathways, process integration, and system-level energy and cost trade-offs.

Though these plants are present in abundance with favorable compositional metrics, they also require tailored deconstruction and conversion protocols, process integration, and cost-energy trade-offs across fuel pathways. For example, switchgrass, despite its broad ecological adaptability (~ 2.3 billion hectares globally) and moderate lignocellulosic composition (≈39.4% cellulose, 20.3% hemicellulose, 21.2% lignin) (Zhang et al. 2012; Bai et al. 2022), delivers ethanol yields of 370–450 gallons per acre, yet its overall system efficiency remains constrained by variable biomass productivity, declining photosynthetic efficiency at maturity, and high pretreatment and logistics costs (Adler et al. 2006; David and Ragauskas 2010; Falls et al. 2011; Haberzettl et al. 2021). In contrast, *Jatropha curcas* follows a lipid-centric conversion logic, where high oil content (30–40%) enables biodiesel production via transesterification, but its biofuel viability depends on effective valorization of lignocellulosic press cake into biogas or soil amendments, as oil extraction alone is insufficient to offset cultivation and processing costs, underscoring the necessity of circular biorefinery integration (Riayatsyah et al. 2022; Milano et al. 2024). Bast fiber crops such as jute and kenaf, characterized by high cellulose (45–65%) and comparatively low lignin (8–15%), present favorable deconstruction profiles for biochemical conversion and polymer precursors; however, their value proposition increasingly lies in co-production of bioplastics and fuels rather than standalone bioenergy, reflecting a strategic shift toward multifunctional biomass utilization (Rowell and Stout 2007; Elfaleh et al. 2023; Turhan-Haskara 2024). High-yield perennial C4 grasses such as Miscanthus (> 20 t ha⁻^1^ yr⁻^1^; 40–50% cellulose, 20–30% hemicellulose, 15–25% lignin) and SRWCs like poplar and willow (40–50% cellulose, 20–30% hemicellulose, 20–25% lignin) offer advantages in land-use efficiency, carbon sequestration, and reduced agronomic inputs, yet their dense lignocellulosic matrices impose persistent pretreatment severity requirements that constrain biochemical routes and shift interest toward thermochemical or hybrid conversion platforms (Lewandowski et al. 2003; Heaton et al. 2004). Bamboo, with rapid maturity (3–5 years) and lignocellulosic composition comparable to woody biomass (40–50% cellulose, 20–30% hemicellulose, 20–25% lignin), exemplifies a high-yield substrate whose bioethanol and biobutanol potential is strongly contingent on pretreatment selectivity and enzymatic efficiency, limiting scalability without advances in low-energy deconstruction (Aizuddin et al*.* 2023; Liang et al. 2023; Ummalyma et al. 2024).

Algae represent a fundamentally distinct, non-terrestrial biofuel paradigm, offering lipid contents up to 70% dry weight and highly variable carbohydrates (3–70%), bypassing land-use competition and enabling CO₂ mitigation (Hannon et al. 2010; Ullah et al. 2014; Sudhakar and Viswanaathan 2019). However, despite theoretical superiority for biodiesel and ethanol co-production, commercialization remains constrained by energy-intensive harvesting, cell disruption, solvent extraction, and tightly controlled cultivation requirements in open ponds or photobioreactors (Das 2016). Collectively, these systems demonstrate that scalable biofuel production requires alignment of feedstock traits with conversion technologies, coproduct valorization, and integrated biorefinery design rather than feedstock-centric optimization alone.

In conclusion, the effective utilization of diverse biomass feedstocks for biofuel synthesis depends on moving beyond feedstock-centric optimization toward integrated, technology-driven biorefinery design. While no single agricultural residue or energy crop is universally optimal, strategic blending of feedstocks with complementary properties can mitigate constraints related to moisture content, inhibitor formation, ash accumulation, and energy demand, thereby improving process stability, plant utilization, and economic resilience. In this context, AI provides a unifying framework to operationalize biomass diversity rather than treat it as a source of variability. By integrating spectroscopic, compositional, and imaging data, AI enables rapid feedstock characterization, real-time sorting and blending, and early identification of recalcitrant traits that impair conversion efficiency. More broadly, AI-driven analytics support dynamic matching of feedstocks to biochemical, thermochemical, or hybrid conversion routes, optimize pretreatment severity, and guide enzyme and microbial platform selection. Together, these capabilities enable adaptive biorefineries that can respond to fluctuating biomass supply while maximizing carbon and energy recovery across multiple fuel pathways. As such, the convergence of feedstock diversity, integrated biorefinery concepts, and AI-enabled decision-making will be central to achieving scalable, resilient, and economically viable biofuel production systems.

## Deconstruction of lignocellulosic biomass into biofuels.

### Pretreatment strategies for releasing carbohydrates from lignocellulosic biomass

**Fig. 3:**
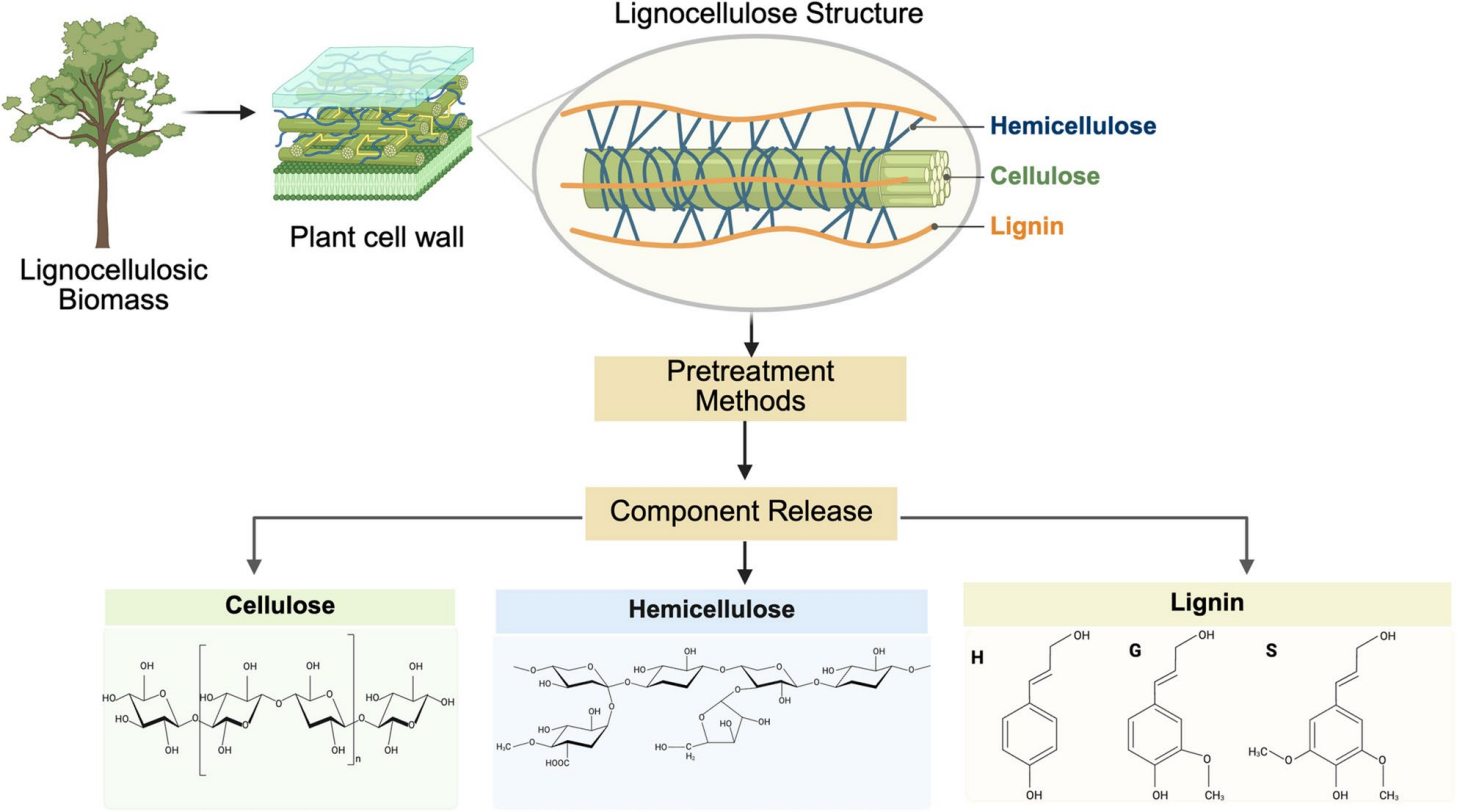
Structural hierarchy and chemical deconstruction of lignocellulosic biomass. This schematic figure illustrates the hierarchical organization of a lignocellulosic plant, transitioning from the macroscale plant structure to the molecular composition of the plant cell wall. The diagram highlights the arrangement of microfibrils, where crystalline cellulose cores are tethered by hemicellulose chains and encapsulated within a lignin matrix. Following various pre-treatment methods, the recalcitrant structure is disrupted to release these primary components, which are further characterized by their chemical structures: cellulose as a linear glucose polymer, hemicellulose as a complex heteropolymer, and lignin as a network of phenolic monomers, specifically* p*-hydroxyphenyl (H), guaiacyl (G), and syringyl (S) units

Despite diversity and abundance of feedstocks, their effective conversion remains constrained primarily by downstream technological limitations, particularly pretreatment methods. In plants, carbohydrates are sequestered within the cell wall as part of a complex and heterogeneous matrix composed of cellulose, hemicellulose, and lignin, the composition of which varies widely among species. For example, softwoods are rich in guaiacyl-type lignin, whereas hardwoods contain both guaiacyl and syringyl units, influencing carbohydrate accessibility (Fig. 3) (Obrzut et al. 2023). Pretreatment is therefore the first and often most technically challenging step in converting lignocellulosic materials into fermentable sugars. Recent studies have focused on developing more sustainable and efficient pretreatment technologies that reduce energy consumption, inhibitor formation, and chemical usage while improving sugar recovery. Deep eutectic solvents (DESs) and advanced ionic liquid systems (ILs) have gained attention as green solvents capable of selectively dissolving lignin and reducing cellulose crystallinity, thereby enhancing enzymatic digestibility and overall biomass fractionation efficiency (Barbará et al. 2025; Riaz et al. 2025a). These solvent systems offer advantages such as tunable physicochemical properties, recyclability, and compatibility with integrated biorefinery concepts. Recent research has also explored hybrid pretreatment strategies, including DES-assisted mechanical refining, microwave-assisted pretreatment, and IL-enzyme integrated systems, which significantly improve delignification and sugar yields while lowering process severity (Liu et al. 2015). Furthermore, advances in computational modeling and ML are enabling predictive optimization of pretreatment conditions by correlating biomass structural features with pretreatment efficiency. These emerging approaches aim to overcome major industrial limitations such as high pretreatment costs, solvent recovery challenges, and variability in biomass composition, thereby accelerating the development of scalable and environmentally sustainable lignocellulosic biorefineries (Riaz et al. 2025b; Woźniak et al. 2025). It disrupts the structural complexity of the biomass and liberates carbohydrates for enzymatic hydrolysis and fermentation (Tables 1 and 2).

**Table 1:** Overview of commonly used pretreatment methods for releasing carbohydrates and their impact on conversion yields

Pretreatment method	Feedstock	Released carbohydrates (post enzymatic hydrolysis)	Biofuel Yield/Conversion Efficiency	Remarks/Advantages	References
DA (H₂SO₄, 0.5–1%, 160–180 °C)	Corn stover	Glucose, xylose	85–90% ethanol yield	High hemicellulose solubilization; generates furfural inhibitors	(Humbird et al. 2011)
Alkali (NaOH, 1–2%, 121 °C)	Rice straw	Glucose, arabinose	0.45 g ethanol/g sugar (≈ 88% of theoretical)	Removes lignin efficiently; improves enzyme accessibility	(Oberoi et al. 2012)
Steam explosion (200 °C, 10 min)	Sugarcane bagasse	Glucose, xylose, arabinose	102 L ethanol/ton dry biomass	Minimal chemical input; partial lignin modification	(MacRelli et al. 2012; Rocha et al. 2012)
CELF (Co-solvent Enhanced Lignocellulosic Fractionation)	Poplar and kraft lignin	High glucose and xylose recovery	High enzymatic digestibility	Efficient lignin removal using THF–water co-solvent system	(Zhao et al. 2022)
RCF (Reductive Catalytic Fractionation)	Hardwood (birch sawdust)	Preserved carbohydrate pulp	High lignin-derived phenolic monomer yield	Lignin-first strategy enabling simultaneous lignin valorization	(Van Den Bosch et al. 2015b)
Organosolv fractionation	Hardwood / Agricultural residues	Glucose (High cellulose recovery)	Improved enzymatic digestibility	Produces high-purity lignin and enhances biomass fractionation	(Ragauskas et al. 2014)
Organosolv (ethanol/water + acid catalyst)	Wheat straw	Glucose, xylose	≈75–85% cellulose conversion; ethanol yields up to ~ 0.45–0.48 g g⁻^1^ fermentable sugars	Generates highly digestible cellulose and a sulfur-free, high-purity lignin co-product suitable for valorization	(Huijgen et al. 2012)
Acid-assisted + enzymatic hydrolysis (H₂SO₄ 1%, 121 °C, followed by cellulase + β-glucosidase)	Coconut husk	Glucose, xylose	0.41 g ethanol/g sugar	High cellulose content; pretreatment crucial to disrupt lignin barrier	Manuscript is in progress
ILs pretreatment	Miscanthus	Glucose, xylose	92% glucose yield; 0.45 g ethanol/g sugar	Excellent delignification, mild conditions	(Auxenfans et al. 2014; Gschwend et al. 2018)
Corn cob	Wet oxidation (O₂, Na₂CO₃)	Glucose, xylose	0.48 g ethanol/g sugar	Removes phenolics; good enzyme accessibility	(Gschwend et al. 2018)

**Table 2:** Mechanistic strategies for enhancing lignocellulose deconstruction

Approach	Key mechanism	Feedstock	Key findings	References
LPMO-Peroxygenase Synergy	Controlled low-dose H₂O₂ feeding enhances LPMO oxidation efficiency and reduces enzyme deactivation	Acid-pretreated wheat straw, spruce pulp	Increased cellulose conversion by 30–40% compared with O₂-only systems	(Stepnov et al. 2022)
Photochemical H₂O₂ Generation (Lignin-Driven)	Visible-light irradiation of lignin generates H₂O₂ and electron donors for LPMO activation	Birchwood lignin + Avicel	Light-exposed lignin increased LPMO-mediated oxidation ~ 3 × vs. dark; improved glucose release	(Kommedal et al. 2023b)
Lignin-First Pretreatment + Photostimulated Hydrolysis	Lignin-first pretreatment produces photoactive lignin fragments enabling light-driven LPMO catalysis	Poplar and spruce biomass	1.6-fold yield increase under visible light; synergy with oxidative lignin fractions	(Magri et al. 2024b)
Engineered LPMO-CBH Co-Expression	Overexpression of *cbh1* and *LPMO* in *Trichoderma reesei* improves real-substrate hydrolysis	Steam-exploded corn stover	25% shorter hydrolysis time, + 18% glucose yield at 20% solids loading	(Ogunyewo et al. 2020b)
Lignin Modification (Electron-Donor Enhancement)	Lignin sulfonation or aromatic additive (2-naphthol) improves electron transfer and enzyme binding	Alkali-treated lignin–cellulose composites	35% higher oxidative cleavage; lower non-productive binding	(Sayler et al. 2024; Yuan et al. 2025a)
AI / Structure-Based LPMO Design	Machine-learning guided mutations to improve catalytic domain stability and Cu-redox turnover	Model substrates (cellulose nanofibrils)	Identified mutations near His-brace that double H₂O₂ tolerance and prolong half-life	(Manikandan and Yennamalli 2025)

As no single pretreatment method is universally applicable, method selection depends on feedstock composition, desired sugar yield, process efficiency, economic considerations, and industrial scalability (Himmel et al. 2007a). Common approaches include physical (e.g., milling and steam explosion), chemical (e.g., acid, alkali, ILs, and organosolv), and physicochemical (e.g., ammonia fiber expansion (AFEX)) methods (Baruah et al. 2018; Bimestre et al. 2022). Each method has limitations, such as high temperature and pressure requirements, equipment corrosion, or the formation of inhibitory compounds like furfural, HMF, phenolics, and organic acids. These inhibitors often require downstream detoxification prior to fermentation, increasing process complexity and wastewater generation (Brodeur et al.2011a) (supplementary Table S1).

Pretreatment chemistry governs not only sugar release and solid loading but also the chemical ecology of the hydrolysate, which directly impacts microbial performance. For instance, Dilute acid (DA) pretreatment primarily solubilizes hemicellulose, producing mainly C5 oligomers but produces inhibitory compounds such as furfural, hydroxymethylfurfural (HMF), and organic acids (Jönsson et al. 2013). In contrast, AFEX pretreatment preserves carbohydrates and generates minimal fermentation inhibitors while enhancing enzymatic digestibility of lignocellulosic biomass (Yang and Charles E. Wyman 2008; Bals et al. 2010; Chundawat et al. 2011). Similarly, ILs and organosolv pretreatment effectively remove lignin under mild conditions, yet leave solvent residues (Brandt et al. 2013a; Nair et al. 2023). Meanwhile, steam explosion partially solubilizes hemicellulose and lignin but produces phenolic inhibitors (Palmqvist and Hahn-Hägerdal 2000a). Furthermore, oxidative or photochemical methods generate reactive oxygen species and oxidized aromatics that can disrupt cell membranes (Balan 2014).

Currently, sequential acid–alkali or alkali pretreatment followed by enzymatic hydrolysis remain the most widely used and cost-effective strategies for industrial bioethanol production (Kim and Kim 2013; Xu and Huang 2014). Acids primarily solubilize and hydrolyze hemicellulose, cleaving lignin carbohydrate linkages and increasing cellulose accessibility (Brodeur et al. 2011b). Alkalis mainly remove and modify lignin, reducing biomass recalcitrance, increasing porosity, and minimizing non-productive enzyme binding (Kim et al. 2016). Future developments are likely to increasingly adopt ILs based pretreatments, which deliver high sugar yields, enhanced cellulose purity, and improved environmental performance at the laboratory scale. ILs effectively disrupt the plant cell-wall matrix by dissolving cellulose and lignin and reducing cellulose crystallinity, thereby markedly improving enzymatic digestibility and sugar release (Hou et al. 2017). Along with deep eutectic solvents, ILs can solubilize major lignocellulosic polymers or even whole biomass and offer advantages such as tunability and reusability. Despite current cost and technical barriers to large-scale deployment, advances in bio-based ILs are improving industrial feasibility (Verdía Barbará et al. 2025). Recent advances in “Lignin-First” biorefinery strategies, particularly reductive catalytic fractionation (RCF)**,** enable the selective depolymerization of lignin into soluble phenolic monomers and dimers while preserving the carbohydrate pulp for further conversion (Van Den Bosch et al. 2015a). This approach has been extensively developed in Europe and further investigated in continuous flow systems for biomass valorization (Anderson et al. 2016, 2017; Li et al. 2025; Sulis et al. 2025) discusses these innovations in depth. ILs represent the most analytically superior conversion platform because they directly disrupt hydrogen bonding in cellulose, solubilize lignin through π-π and electrostatic interactions, and reduce cellulose crystallinity without relying on severity-driven bond cleavage, resulting in substrates with maximal enzymatic accessibility and minimal carbohydrate loss (Brandt et al. 2013b; Hou et al. 2017). Unlike acid–base systems, ILs decouple deconstruction efficiency from inhibitor formation, enabling higher solid loadings, reduced enzyme dosages, and improved mass-transfer kinetics. However, their deployment is constrained by solvent viscosity, energy-intensive recovery, residual ILs toxicity to enzymes and microbes, and capital costs associated with solvent handling (Verdía Barbará et al. 2025). A recent review (Li et al. 2025; Sulis et al. 2025) discusses these innovations in depth.

AI and ML are increasingly enabling predictive, data-driven optimization of lignocellulosic pretreatment. Models such as artificial neural networks (ANN), random forests, and support vector machines integrate biomass structural and compositional features, including cellulose crystallinity, lignin content, and S/G lignin ratio, ash, and moisture content derived from experimental and spectroscopic datasets (FTIR/NMR). These approaches have improved optimization of DA, alkaline, steam explosion, organosolv, ILs, and deep eutectic solvent pretreatments by identifying optimal severity factors, temperatures, and solvent conditions while minimizing inhibitor formation and carbohydrate loss (Vuong et al. 2025; Zhou et al. 2025). Overall, AI-guided frameworks support the rational co-design of pretreatment and biological conversion strategies, reducing trial-and-error and accelerating scalable, low-impact lignocellulosic biorefineries (Liao and Yao 2021b).

Emerging conceptual frameworks classify pretreatments as oxidative, reductive, phenolic-rich, or salt/solvent-dominant chemistries. Each approach generates a distinct chemical environment, shaped by the nature of the feedstock, that influences microbial tolerance and fermentation performance. This ecological perspective reframes pretreatment not simply as a physicochemical operation but as a biological conditioning step that ultimately determines the success of downstream microbial biofuel production (Zhou et al. 2023).

### Enzymatic deconstruction of lignocellulosic biomass for biofuel production

While physical and chemical pretreatments remain essential for biomass opening and lignin disruption, enzymatic deconstruction of pretreated biomass is preferred for efficient, scalable, and biologically compatible conversion of lignocellulose into fermentable sugars (Sun and Cheng 2002; Mosier et al. 2005a). Unlike harsh chemical or thermal treatments, enzymatic hydrolysis proceeds under mild conditions, minimizes sugar degradation, and limits the formation of inhibitory byproducts that impair microbial fermentation (Tables 3 and 4). Enzymatic deconstruction bridges pretreatment and microbial biofuel synthesis, enabling high yields and sustainable, integrated processes. Its substrate specificity ensures efficient sugar recovery for downstream conversion and is compatible with fermentation and advanced biorefinery strategies, including consolidated bioprocessing (CBP), where enzyme production, biomass hydrolysis, and fermentation occur simultaneously within a single integrated process (Olson et al. 2012). Coordinated action of cellulases, hemicellulases, and other carbohydrate-active enzymes makes it a cornerstone of advanced biofuel production (Stepnov et al. 2022a).

**Table 3:** Comparative overview of enzyme systems for advanced lignocellulose saccharification

Enzyme	Reaction mechanism	Cleavage bond	Cofactor/activation requirement	Representative findings	References
Cellulases (Endoglucanase, Exoglucanases, β-Glucosidase)	Hydrolytic cleavage via acid–base catalysis	β-1,4-glycosidic bonds in amorphous and semi-crystalline cellulose	None (classical hydrolases)	Canonical enzymes for cellulose depolymerization; limited activity on crystalline regions; synergistic with LPMOs	(Ejaz et al. 2021)
Redox-assisted (oxidative enzymatic)	Sulfite pulping, controlled H₂O₂ dosing during saccharification	Controlled redox species; reduced lignin recalcitrance	Cellulose-enriched with accessible hemicellulose	Enables LPMO-assisted cellulase systems; enhanced saccharification under controlled redox conditions	(Costa et al. 2020b; Stepnov et al. 2022)
Peroxygenases (Unspecific or Aryl-alcohol peroxygenases)	H₂O₂-driven monooxygenation and epoxidation; lignin oxidation	Aromatic and aliphatic C–H or C = C bonds (lignin fragments)	H₂O₂; heme or flavin cofactor	Catalyze oxidative lignin modification, producing intermediates that sustain LPMO turnover	(Yuan et al. 2025b)
Photochemical Redox Systems (Lignin-mediated)	Light-driven generation of reactive oxygen species (mainly H₂O₂) via photoexcited lignin chromophores	Indirect activation of LPMOs, enhancing oxidative cellulose cleavage	Visible light; lignin; oxygen and water	Visible-light-exposed lignin generates in situ H₂O₂, driving LPMO activity and cellulose solubilization	(Kommedal et al. 2023)
Engineered Enzyme Cocktails (LPMO + CBH1 Overexpression)	Combined oxidative and hydrolytic depolymerization with endogenous electron supply	Crystalline cellulose and mixed hydrolysates	Intrinsic electron transfer from lignin phenolics; no external donors required	Engineered fungal strains achieve superior saccharification at 20% solids loading	(Ogunyewo et al. 2020)
AI- and Structure-Guided LPMO Design	Rational / ML-guided engineering, QM/MM active-site optimization	Tuned Cu–O intermediates; controlled H₂O₂ turnover	Oxidative cellulose fragments	Designed LPMOs show improved stability, Cu-redox kinetics, controlled peroxygenase reactivity	(Beeson et al. 2011; Hemsworth et al. 2013; Yang, Wu and Arnold, 2018; Yang et al. 2024)

**Table 4:** Recent advances in enzyme redox systems for enhanced biomass saccharification

Pretreatment class	Method	Dominant chemical environment & inhibitors	Typical sugar composition (after pretreatment)	Optimal enzyme or microbial strategy	References
Oxidative	Alkaline H₂O₂, ozonolysis, wet oxidation	High oxidant load (H₂O₂, ROS); moderate phenolics	Glucose-rich (> 65%) and, moderate xylose (20–30%)	LPMO-peroxygenase synergy; H₂O₂-tolerant cellulases; oxidative stress–resistant microbes (*Z. mobilis*, *Trichoderma reesei*)	(Schmidt and Thomsen 1998; Ullah et al. 2023)
Reductive	Sodium dithionite, sulfide-based, bio-reductive systems	Reductive sulfur species; lignin depolymerization intermediates; low phenolic content	Balanced glucose/xylose (~ 1:1)	Anaerobic fermenters (Clostridium spp.); Redox-balanced enzyme cocktails with NADH regeneration modules	(Brienza et al. 2021; Stepnov et al. 2024)
Phenolic-Rich	Steam explosion, DA, AFEX	High concentrations of ferulic, vanillic, and p-coumaric acids; furan aldehydes (HMF, furfural)	Glucose(40–50%) xylose (25–35%) Residual lignin-bound sugars	Laccase-LPMO systems for detoxification and oxidative cleavage; engineered *Saccharomyces cerevisiae* or *Pichia stipitis* strains tolerant to phenolics	(Palmqvist and Hahn-Hägerdal 2000b; Jönsson and Martín 2016)
Salt / Residual Solvent	ILs (imidazolium-, cholinium-based), organosolv	Residual salts, acetate/ethanol traces, ionic solvent toxicity	Glucose(50–60%) xylose (20–25%) lignin 10–15%	Halotolerant microbes (*Halomonas*, *Marinobacter*) or IL-tolerant *E. coli*; cellulases engineered for solvent stability (via ML-guided redesign)	(Zhao 2010; Brandt et al. 2013b)
Acidic Hydrolytic	DA H₂SO₄, HCl, H₃PO₄	Organic acids, furfural, HMF, residual sulfate	High pentose yield (xylose 40–60%) with glucose (30–40%)	Xylanase-rich cocktails, acidophilic LPMOs, and engineered *Z. mobilis* expressing *xylose isomerase /xylulo kinase* pathways	(Jensen et al. 2010)
Photochemical/Advanced Oxidative	UV–H₂O₂, TiO₂, photocatalytic lignin oxidation	Reactive oxygen species, quinone intermediates; low furan	High glucose recovery (~ 70%) with moderate lignin oxidation	Photoactivated LPMO-peroxygenase coupling; artificial photosensitizers for controlled redox activation	(Costa et al. 2020b; Stepnov et al. 2022)

Cellulases form the central catalytic framework of biomass-degrading enzyme systems and act through a tightly coordinated synergistic mechanism. This system comprises three principal enzyme classes: (i) endoglucanases, which randomly cleave internal β-1,4-glycosidic linkages within cellulose chains to generate new chain ends, (ii) exoglucanases (cellobiohydrolases) processively release cellobiose from crystalline cellulose, and (iii) β-glucosidases alleviate product inhibition by converting cellobiose to glucose (Stepnov et al. 2022a). Commercial enzyme cocktails, largely derived from fungi such as *Trichoderma reesei* and *Aspergillus niger*, have been optimized for industrial application, yet the ordered structure of cellulose still necessitates effective pretreatment and auxiliary enzymatic support (Stepnov et al. 2022b; Angeltveit et al. 2024).

In addition to cellulose, hemicellulose accounts for approximately 20–35% of plant biomass and is highly heterogeneous in composition and structure (Anwar et al. 2014). Its efficient depolymerization, therefore, requires a diverse suite of hemicellulases. Key enzymes include xylanases, mannanases, arabinofuranosidases, and acetyl xylan esterases, which act on branched polysaccharides such as xylan and glucomannan (Shallom and Shoham 2003). The coordinated action of these enzymes releases pentose sugars, primarily xylose and arabinose, that can be converted to biofuels by engineered microbial strains that are capable of pentose fermentation (Østby and Várnai 2023).

The discovery of Lytic Polysaccharide Monooxygenases (LPMOs) has markedly advanced lignocellulose bioconversion by introducing oxidative cleavage of glycosidic bonds in crystalline cellulose using oxygen or hydrogen peroxide and external electron donors (Vaaje-Kolstad et al. 2010; Phillips et al. 2011; Kommedal et al. 2023a). By disrupting cellulose crystallinity and generating new chain ends, LPMOs enhance cellulase activity and reduce enzyme loading and process costs (Magri et al. 2024b; Costa et al. 2020). This oxidative mechanism, particularly cleavage at the C1 and C4 positions of cellulose, represents one of the most impactful recent advances in biomass conversion, substantially improving hydrolysis rates. However, the copper-dependent redox chemistry of LPMOs introduces new operational challenges, including the need for precise oxygen and hydrogen peroxide control and mitigation of reactive oxygen species, which can compromise enzyme stability and negatively affect downstream fermentation (Bissaro et al. 2017; Kommedal et al. 2023a).

In parallel, auxiliary enzymes such as pectinases facilitate the deconstruction of pectin-rich biomass, such as fruit residues and agricultural wastes, by dismantling the homogalacturonan and rhamnogalacturonan networks that restrict polysaccharide accessibility. Lignin-modifying enzymes further enhance biomass deconstruction by altering lignin structure and porosity. For example, laccases catalyze one-electron oxidation of phenolic lignin units, promoting cleavage or rearrangement of β-O-4 aryl ether bonds and condensed C–C linkages (β-β, β-5, 5–5), as well as oxidation of phenolic hydroxyl groups. Class II peroxidases, including lignin peroxidase and manganese peroxidase (Qin et al. 2014; Janusz et al. 2017; Cai et al. 2023), employ hydrogen peroxide to oxidatively attack non-phenolic lignin structures, targeting recalcitrant β-O-4 ether linkages and generating aromatic radical intermediates that drive lignin depolymerization and side-chain cleavage (Wariishi et al. 1991; Hammel and Cullen 2008). Together, pectinases, laccases, and peroxidases weaken lignin–carbohydrate complexes, loosen the cell wall matrix, and improve cellulase and hemicellulase penetration, thereby significantly enhancing enzymatic saccharification under mild pretreatment conditions (Tarasov et al. 2018).

Recent advances in protein engineering, directed evolution, and synthetic biology have enabled the development of a thermostable, pH-tolerant, and substrate-specific cocktail of enzymes tailored for industrial conditions (Ogunyewo et al. 2020a). Structural and spectroscopic analyses, including carbohydrate-binding modules characterization and electron paramagnetic resonance studies, have clarified enzyme–substrate interactions and guided domain engineering to enhance cellulose accessibility, catalytic efficiency, and substrate affinity (Himmel et al. 2007b). Computational and AI/ML approaches, such as ANN and random forest models, are increasingly applied to optimize enzymatic hydrolysis and accurately predict ethanol yield (R^2^ ≈ 0.96) (Smuga-Kogut et al. 2021). Molecular simulations and AI-guided design have also accelerated optimization of LPMOs’ performance, particularly their copper redox chemistry and hydrogen peroxide management (Bissaro et al. 2017).

Nanotechnology-based immobilization and nanobiocatalyst platforms further enhance enzyme stability, activity, and reusability under harsh conditions like pH and oxidative stress (Velo-Gala et al. 2022). Immobilization on engineered nanomaterials, including magnetic nanoparticles, mesoporous silica, carbon nanotubes, and metal organic frameworks, improves enzyme conformational rigidity while preserving active-site accessibility, thereby reducing denaturation and proteolytic degradation (Sheldon and van Pelt, 2013). Core–shell nanocomposites, alginate-chitosan microcapsules, and polymer-inorganic hybrids enable controlled microenvironments around enzymes, facilitating enhanced mass transfer, tunable redox or pH buffering at the nanoscale, These systems also extend enzyme half-life, improve adaptability to process conditions, and allow repeatedreuse" instead of "and extending enzyme half-life, adapt to process conditions, and allow repeated reuse (Taqieddin and Amiji 2004; Chandel et al. 2022).

These innovations support robust CBP by allowing simultaneous hydrolysis and fermentation and have been translated into commercial enzyme formulations such as Cellic® CTec (Jain et al. 2019), Accelerase® (Advanced BioFuels USA – Genencor Introduces Accellerase® DUET, 2010), Rohapect® (Bustamante-Vargas et al. 2015), for efficient biomass deconstruction at large scale. Collectively, these advances position enzymatic deconstruction as a core component of industrial biofuel production, enhancing efficiency, scalability, and sustainability in next-generation lignocellulosic biorefineries.

## Microbial conversion of sugars into bioenergy compounds

Following feedstock pretreatment and enzymatic deconstruction, microbial conversion of released sugars represents the final and decisive step in the biofuel production pipeline. This stage requires the selection of robust microbial strains capable of tolerating and metabolizing inhibitor-rich hydrolysates, which is critical for cost-effective bioenergy production. Microbial conversion strategies broadly include single engineered strains, sequential cultures, microbial consortia, and cell-free biocatalysis (Bergquist et al. 2020; Cho et al. 2022). Single-chassis systems rely on inhibitor-tolerant strains capable of fermenting mixed sugars (Parreiras et al. 2014; Lama et al. 2024), while microbial consortia distribute detoxification and metabolic functions across species, improving stability and substrate utilization (Zuroff and Curtis 2012; Minty et al. 2013). Cell-free systems bypass cellular toxicity and offer precise control and enhanced tolerance to inhibitory molecules, though scalability remains challenging (Dudley et al. 2015; Korman et al. 2017).

Microbial performance is strongly influenced by pretreatment-specific inhibitor profiles. For example, corn stover typically yields furan-rich hydrolysates, sugarcane bagasse produces mixed furan phenolic inhibitors, and hardwoods generate phenolic-rich streams (Parawira and Tekere 2011). To address this variability, microbial tolerance strategies generally integrate three complementary modules: (i) tailored enzymatic detoxification of targeted aldehydes and phenolics, (ii) redox cofactor balancing via enhanced NAD(P)H regeneration, and (iii) membrane and efflux engineering, including transporter overexpression and membrane reinforcement (Chang et al. 2018; Ujor and Okonkwo 2022; Asefi et al. 2024). Tailored combinations of these modules have delivered 10–30% improvements in fermentation rates and ethanol yields, with furan-rich hydrolysates responding best to reductive detoxification and phenolic-rich hardwood streams benefiting from oxidative enzymatic treatments and strengthened membrane systems (Plácido and Capareda 2015; Artifon et al. 2018). Metabolic engineering and adaptive laboratory evolution (ALE) are commonly employed to enhance resistance, particularly through overexpression of detoxification enzymes that convert furans into less toxic alcohols and restore redox balance (Jilani and Olson 2023; Yao et al. 2023; Lama et al. 2024).

*Escherichia coli* and *Saccharomyces* spp. (yeast) have long been the dominant microbial platforms for bioenergy conversion and have undergone extensive metabolic and genetic modification to improve product yield and robustness. However, the highly optimized and tightly regulated metabolic networks of these model organisms increasingly limit further engineering flexibility, particularly under the complex conditions imposed by lignocellulosic hydrolysates (Lee et al. 2011; Trinh et al. 2011). This has driven a strategic shift toward non-model but naturally efficient biofuel-producing microbes, including *Zymomonas mobilis*, *Clostridium* spp., and engineered cellulolytic consortia (Minty et al. 2013; Mishra et al. 2025a). Among these, *Z. mobilis* is a natural ethanologen with a compact genome and an energy-efficient Entner-Doudoroff (ED) pathway, enabling high ethanol yields with reduced biomass formation and comparatively lower engineering complexity than larger-genome systems such as *E. coli* and yeast. Its facultative anaerobic physiology and capacity for aerobic fermentation further distinguish it from conventional anaerobic fermenters, although its narrow substrate spectrum and sensitivity to pretreatment-derived inhibitors constrain broader deployment (Yang et al. 2018; Mishra et al. 2025b). In parallel, CBP organisms, particularly anaerobic *Clostridium* species and engineered cellulolytic consortia, offer a complementary advantage by integrating enzyme production, biomass saccharification, and fermentation within a single biological system, thereby reducing pretreatment and hydrolysis costs despite trade-offs between growth, enzyme secretion, and product formation efficiency (Lynd et al. 2002, 2017b; Kung et al. 2012; Olson et al. 2012). Beyond sugar fermentation, *Novosphingobium aromaticivorans* represents a distinct functional chassis that, while strictly aerobic and non-fermentative, efficiently degrades lignin-derived aromatic compounds through oxidative pathways, facilitating lignin valorization and detoxification in hybrid biorefinery configurations (Perez et al. 2021; Mishra et al. 2025b) (Table 5). Collectively, these platforms underscore that no single microorganism currently exhibits broad tolerance and metabolic compatibility with diverse lignocellulosic hydrolysates, highlighting the necessity for systems-level metabolic reprogramming and hybrid microbial strategies to enable flexible, feedstock-agnostic biorefineries. Working with non-model microorganisms remains challenging due to the limited availability of species-specific genetic toolkits, incomplete or poorly curated genome annotations, and the absence of well-characterized, laboratory-domesticated strains. These constraints hinder predictable gene expression, efficient genome editing, and reproducible phenotypic outcomes (Yan et al. 2024). To overcome these barriers, several strategies have been developed, including the expansion of broad-host-range vectors, development of modular synthetic biology toolkits (Lal et al. 2019, 2021; Mishra et al. 2025a), application of transposon mutagenesis (Myers et al*.* 2022; Eckmann et al. 2025), and CRISPR-based editing systems adapted for non-model hosts (Eckmann et al. 2025).

**Table 5:** Microbial Platforms for the valorization of phenolic substrates and lignin derivatives

Microbes	Phenolic substrate	Valuable products	Key enzymes	References
*Aspergillus niger*	Phenolic acids from citrus waste	Antioxidant phenolic acids (ferulic, p-coumaric acids)	Esterases, phenolic acid decarboxylase	Kumar et al. (2025)
*Trametes* spp.* (white-rot fungi)*	Lignin-derived phenolic compounds (e.g., guaiacol, syringol, vanillin, ferulic and *p*-coumaric acids)	Detoxified hydrolysates, low-molecular-weight aromatic acids and aldehydes (e.g., vanillic acid), and modified lignin fragments suitable for downstream bioconversion	Laccase, manganese peroxidase (MnP), lignin peroxidase (LiP)	Baldrian (2006)
*Lactobacillus* spp.	Phenolic acids, flavonoids	Bioactive phenolics with antioxidant and antimicrobial properties	Phenolic acid reductase, glycosidase	(Zhou et al. 2020; Wang et al*.* 2023a)
*Bacillus* spp.	Lignocellulosic phenolics	Biopolymers, antioxidants, flavor compounds	Laccase, peroxidase, reductases	(Wang et al. 2021, 2023b; Katagi et al. 2023)
*Streptomyces tunisiensis*	Ferulic acid	4-vinyl guaiacol, acetovanillone	Ferulic acid-converting enzymes	(Lu et al. 2025)
*Saccharomyces cerevisiae*	Flavonoid glycosides	Flavonoid aglycones (bioactive molecules)	β-glucosidase	(Schmidt et al. 2011)
*Pseudomonas putida*	Lignin-derived phenolics	Vanillin, muconic acid	Laccase, monooxygenases, dioxygenases	(H. Wang et al. 2022; Zhang et al. 2024)
*Novosphingobium aromaticivorans*	Guaiacyl, syringyl, p-hydroxyphenyl lignin monomers and β-O-4 aromatic dimers	2-pyrone-4,6-dicarboxylic acid, cis,cis-muconic acid, protocatechuic acid	Lig dehydrogenases, O-demethylases (DesA, LigM), ring-opening dioxygenases (LigAB)	(Cecil et al. 2018)

Although fermentable sugars derived from lignocellulosic biomass serve as the primary substrates for microbial biofuel production, other biochemical feedstocks also play important roles in bioenergy systems. Lignocellulosic biomass contains several carbohydrates, among which glucose and xylose are the most abundant, along with other sugars such as arabinose and cellobiose. An efficient microbial system should ideally utilize most of these sugars and convert them into biofuels. In addition to carbohydrate-based substrates, lipid-based feedstocks have also gained increasing attention in microbial biorefineries, particularly for biodiesel production. Biodiesel is commonly produced through the transesterification of triglycerides derived from vegetable oils, waste cooking oils, animal fats, and microbial lipids. Several microorganisms, including oleaginous yeasts such as *Yarrowia lipolytica*, *Rhodosporidium toruloides*, and *Lipomyces starkeyi*, are capable of accumulating intracellular lipids that can be converted into fatty acid methyl esters (FAMEs), the primary components of biodiesel (Ratledge and Wynn 2002; Li et al. 2008; Beopoulos et al. 2009; Sitepu et al. 2014; Shields-Menard et al. 2018). These microorganisms can utilize diverse carbon sources such as glycerol, fatty acids, and lipid-rich agro-industrial wastes for lipid biosynthesis. The selection and optimization of microbial systems capable of utilizing diverse substrates are crucial for improving biofuel yields and developing efficient integrated biorefinery platforms.

Advances in systems biology, multi-omics, AI-guided modeling, and CRISPR-based engineering are accelerating the development of feedstock-adapted microbes as comprehensively discussed in the referenced studies (Caspeta et al. 2015; Yang et al. 2016; Mishra et al. 2025a). Despite this progress, the sustainable conversion of all sugar monomers presents in agricultural waste and the inhibitory effects of lignin-derived aromatics remain major challenges. To improve overall process sustainability, strategies that generate multiple value-added products alongside biofuels are increasingly being adopted. For example, microbial consortia comprising *Pseudomonas*, *Rhodococcus*, and *Novosphingobium* have demonstrated enhanced degradation of lignin-derived aromatics and improved resource utilization (Xu et al. 2019). Beyond ethanol, these microbes can valorize aromatic compounds into high-value chemicals, with consortia enabling cross-feeding and functional specialization. Recent studies also highlight the use of microbes in microbial fuel cells, where organic substrates are oxidized, and electrons are transferred to electrodes, enabling the simultaneous production of biofuels, bioelectricity (Hoang et al. 2022), organic acids, and biopolymers (J.; Wang et al. 2022; Dakal et al. 2025). Collectively, these approaches increasingly rely on AI-driven optimization of process strategies, including fed-batch and continuous fermentation as well as in situ detoxification, to further mitigate inhibitor effects and enhance system performance (Branska et al. 2024). Overall, integrating AI-guided microbial engineering, adaptive evolution, co-culture design, and advanced process optimization is key to sustainable and economically viable biofuel production from lignocellulosic residues.

## Process integrative technologies

Process integrative technologies play a critical role in advancing biofuel production efficiency, with CBP and electro-fermentation, representing promising approaches (Linger and Darzins 2013; Banner et al. 2021a; Periyasamy et al. 2023). CBP integrates enzyme production, biomass saccharification, and fermentation within a single microbial system, reducing enzyme costs and process complexity. Microorganisms such as *Clostridium thermocellum*, which can both depolymerize lignocellulose and produce ethanol, serve as one of the model CBP platforms (Mbaneme-Smith and Chinn 2015). Thermophilic CBP further exploits heat-tolerant microbes to accelerate reaction kinetics, improve substrate solubility, and minimize contamination risks, enabling efficient conversion at elevated temperatures (Banner et al. 2021b).

Electro-fermentation integrates microbial metabolism with electrochemical inputs, allowing external control of intracellular redox balance and thereby enhancing the production of reduced bioenergy compounds (Namboonlue et al. 2025; Pilania et al. 2025). Although still at an early stage, this approach offers unique opportunities to link biological and electrochemical energy conversion. Collectively, these integrative platforms promise more cost-effective, scalable, and feedstock-flexible biofuel production with reduced reliance on external enzymes and chemical catalysts.

## Artificial intelligence and machine learning for next-generation biorefineries

AI and ML are increasingly central to the design and operation of next-generation biorefineries, offering powerful data-driven tools to manage the complexity of biomass conversion. By enabling predictive modeling, automation, and real-time optimization, AI/ML approaches are improving efficiency across the entire biorefinery value chain, including feedstock handling, pretreatment, and fermentation (Namboonlue et al. 2025), downstream processing, and system-level integration across the biorefinery value chain (Liao and Yao 2021a).

In feedstock characterization and pretreatment, ML models, particularly ANN, effectively capture the heterogeneity arising from biomass type, geography, and processing chemistry. These models show strong predictive capability for estimating fermentable sugar yields, inhibitor formation, and phenolic release during pretreatment, while accounting for nonlinear interactions across pretreatment severity, enzymatic hydrolysis, and downstream valorization (Namboonlue et al. 2025). By integrating multivariate inputs from spectroscopy, multi-omics analyses, and process sensors, ML algorithms identify optimal operating parameters such as temperature, residence time, and reagent concentration, reducing trial-and-error experimentation, energy demand, and inhibitor generation. Recent applications to hydrothermal carbonization report R^2^ values > 0.88 for predicting hydrochar properties, supporting rational process design (Xie and Fan 2025). Similarly, ANN and random-forest models applied to ILs pretreated lignocellulose accurately predicted bioethanol yields (R^2^ ≈ 0.96) and enabled achievement of 84–92% of theoretical ethanol yields under optimized conditions (Smuga-Kogut et al. 2021)**.**

In fermentation systems, AI-driven modeling accelerates process optimization by predicting microbial performance, nutrient requirements, stress responses, and product yields with high accuracy (Wang et al. 2025). Approaches such as ANN, adaptive network-fuzzy inference systems (ANFIS), and response surface methodology (RSM) have been widely applied to optimize fermentation of lignocellulosic substrates, resulting in substantial improvements in bioethanol and biodiesel yields, often reaching 84–98% of theoretical maxima while minimizing inhibitor effects (Awogbemi and Kallon 2023; Owusu and Marfo 2023). AI also supports adaptive process control by integrating real-time sensor data to anticipate deviations and automatically adjust fermentation parameters. In parallel, hybrid AI-mechanistic frameworks that couple machine learning with genome-scale metabolic models have enhanced strain engineering and fermentation of corn stover and sugarcane bagasse by identifying key genetic targets, optimizing metabolic fluxes, and achieving 90–97% of theoretical fuel yields (Cheng et al. 2023; Garg et al. 2025). Collectively, these integrated approaches are transforming microbial bioprocessing for the sustainable production of biofuels, biochemicals, and biopolymers (Table S7).

Downstream processing, traditionally one of the most energy-intensive stages of biorefineries, also benefits from AI-based optimization. Applications include predictive modeling of membrane performance, enhanced control of distillation columns, and optimized solvent or adsorbent selection for purification. Although research in this area is still emerging, early results indicate significant potential for reducing operational costs and improving separation efficiency (Mondal et al. 2023). At the systems level, AI-driven tools for logistics planning, supply chain management, and digital-twin simulations are increasing the resilience and sustainability of biorefineries by reducing feedstock variability and lowering the overall carbon footprint (Egbuna et al. 2025).

**Fig. 4:**
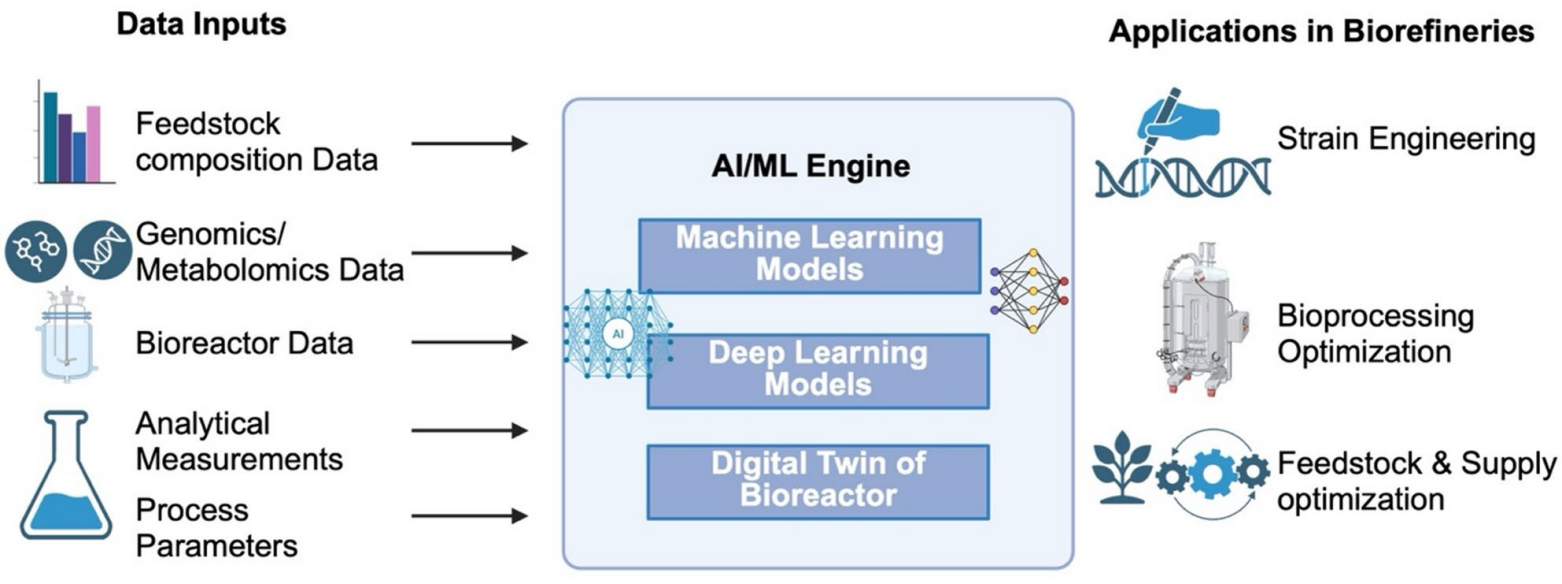
Integration of AI/ML into biorefinery workflows. The figure illustrates the integration of an AI/ML engine into biorefinery workflows, illustrating the flow from multifaceted data inputs to high-value applications in biorefineries. The process begins with the collection of diverse datasets, including feedstock composition, genomics, metabolomics, bioreactor performance, analytical measurements, and process parameters. These inputs are processed by a central computational hub comprising machine learning models, deep learning models, and a digital twin of the bioreactor to stimulate and predict system behavior. Resulting insights derive critical downstream advancements such as strain engineering, bioprocessing optimization, and the streamlining of feedstock and supply chain management, ultimately enhancing the efficiency and scalability of bio-based production

AI is further accelerating progress in carbon capture–linked biofuel production by enabling high-throughput screening of CO₂ adsorbents and catalysts, optimizing integration of carbon capture with hydrogen production, and improving catalytic CO₂ conversion pathways through reactor modeling and LCA (Priya et al. 2023). Collectively, these advances underscore the transformative role of AI and ML in building efficient, adaptive, and sustainable biorefineries for future bioenergy and bio-based manufacturing systems (Fig. 4).

## Recent advances in carbon capture linked biofuels

Carbon capture-linked biofuel production has emerged as a promising pathway toward carbon–neutral and even carbon-negative energy systems. While conventional biofuels reduce dependence on fossil resources, they still release CO₂ upon combustion. The integration of carbon capture and utilization (CCU) strategies addresses this limitation by recycling emitted or captured CO₂ into new biofuels, thereby establishing a circular carbon economy (Ghiat and Al-Ansari 2021).

Recent advances have focused on the development of engineered microbes and photosynthetic platforms capable of directly converting CO₂ into biofuel precursors. Cyanobacteria, microalgae, and metabolically engineered bacteria such as *Cupriavidus necator* are being optimized to channel captured CO₂ into ethanol, butanol, and lipid-based fuels through native and synthetic carbon-fixation pathways (Savakis and Hellingwerf 2015; Singh and Dhar 2019; Panich et al. 2021; Li and Yao 2024). Improvements in photobioreactor design, light distribution, nutrient recycling, and gas–liquid mass transfer have significantly enhanced algal productivity (Kumar et al. 2025). Moreover, coupling algal cultivation systems with industrial flue gases has demonstrated feasibility at the pilot scale, offering simultaneous carbon mitigation and fuel production (Li et al. 2025a).

Parallel progress in materials science has strengthened the capture and conversion components of CCU-linked biofuel systems. Advanced catalytic nanomaterials, such as Cu-based catalysts (Li et al. 2025b) and perovskites (Tian et al. 2024), along with next-generation CO_2_ sorbents such as metal–organic frameworks (Gebremariam et al. 2023), ILs (Pandya et al. 2024), and hybrid adsorbents, have improved CO₂ capture and conversion efficiency, while lowering regeneration energy and operational costs. Increasingly, AI- and ML-driven approaches are being applied to accelerate catalyst discovery, optimize reactor configurations, and enable digital-twin–based process integration. These tools have been reported to improve overall system efficiency by approximately 10–30% through real-time monitoring and optimization (Ghafarian Nia et al. 2025).

Another rapidly advancing area is microbial electrosynthesis, in which electroactive microorganisms use renewable electricity to reduce CO₂ into fuels and value-added chemicals. When integrated with bioenergy with carbon capture and storage (BECCS), such systems offer the potential for net-negative emissions by combining renewable fuel production with permanent CO₂ removal (Rabaey and Rozendal 2010; Kiely et al. 2011).

Collectively, these developments highlight the growing maturity of carbon capture-linked biofuels as a viable climate mitigation strategy. By uniting advances in synthetic biology, catalysis, electrochemical systems, and process engineering, CCU-integrated biofuel platforms offer a dual benefit of greenhouse gas reduction and sustainable energy generation, positioning them as key contributors to the global transition toward a low-carbon economy.

## Techno-economic analysis and life cycle assessment for sustainable process design

TEA and LCA are essential and complementary tools for sustainable bioprocess and bioenergy system design, enabling the simultaneous evaluation of economic feasibility and environmental performance across the entire value chain. TEA systematically estimates capital investment, operating costs, product yields, and minimum selling prices, thereby identifying cost drivers, process bottlenecks, and scale-up challenges in emerging biofuel and biorefinery technologies (Liang et al. 2024). Despite significant technological progress**,** scaling up biofuel production from laboratory to industrial scale remains a major challenge. Large-scale operations must address several technical barriers, including high pretreatment energy requirements, enzyme costs, fermentation inhibition caused by lignocellulosic hydrolysate-derived compounds, and inefficient downstream product recovery (Mosier et al*.* 2005b; Humbird et al. 2011). In addition, process integration, reactor design, mass transfer limitations, and microbial robustness under industrial conditions significantly influence large-scale productivity. Economic challenges are equally critical, as feedstock logistics, pretreatment infrastructure, and enzyme loading contribute substantially to operational costs, often limiting commercial competitiveness with fossil fuels (Cherubini and Strømman 2011a; Lynd et al. 2017b). Furthermore, variability in biomass composition across geographic regions complicates process standardization and stable industrial performance. Addressing these challenges requires integrated strategies that combine advanced strain engineering, process intensification, improved pretreatment technologies, and optimized biorefinery integration, and these strategies are increasingly evaluated using TEA and LCA frameworks (S. N. Naik et al. 2010). In parallel, LCA quantifies the environmental impacts associated with feedstock production, pretreatment, conversion, and downstream processing, using standardized indicators such as greenhouse gas emissions, energy demand, water footprint, and eutrophication potential, and waste generation across the entire production chain, following internationally recognized frameworks (ISO 14040/14044) (Mahmud et al. 2021). Integration of advanced wastewater treatment, detoxification strategies, and by-product valorization effectively reduces chemical oxygen demand and downstream environmental burdens associated with biofuel production (Venkata Mohan et al. 2016). Implementing TEA and LCA at early stages of process design enables quantitative evaluation of trade-offs between capital and operating costs, energy efficiency, and environmental performance, thereby guiding informed decisions on feedstock selection, process configuration, and scale-up strategies (Humbird et al. 2002; Appels et al. 2008; Cherubini and Strømman 2011b). Numerous TEA-LCA studies demonstrate that biorefinery-based process integration and circular waste management approaches improve economic viability while simultaneously lowering greenhouse gas emissions and resource footprints, supporting the development of environmentally sustainable and commercially competitive biofuel production systems (Cherubini and Strømman 2011a; Yang et al. 2018; Pérez-Almada et al. 2023). Recent integration of AI and ML with TEA-LCA further enhances optimization of process parameters and scale-up scenarios, accelerating the development of cost-effective, low-carbon biorefineries aligned with circular bioeconomy goals (Giacomella 2021).

## Conclusions

This review underscores the importance of integrating diverse feedstocks, tailored pretreatment strategies, advanced enzyme systems, and optimized microbial fermentation platforms in modern biofuel production. Biomass-to-biofuel conversion is a highly interdependent, multi-step process in which coordinated optimization is essential to enhance yields, lower costs, and improve sustainability. For example, utilizing diverse feedstocks enhances supply chain flexibility, resilience, promotes efficient land use, and supports local circular economies. Tailored pretreatments considering feedstock-specific chemical ecologies improve enzymatic accessibility, while advanced enzyme systems, like cellulases, hemicellulases, and oxidative enzymes, enable efficient lignocellulose deconstruction. Optimized microbial fermentation using optimized strains, synthetic consortia, and cell-free systems achieve high-yield biofuel conversion even under inhibitory conditions. Coupling these biological processes with carbon capture, electro-fermentation, and CBP enhances carbon efficiency. Supported by AI-driven optimization, multi-omics, and metabolic modeling, these integrated approaches provide a pathway toward scalable, carbon-negative biofuel production and a resilient, sustainable bioeconomy.

## Electronic Supplementary Material

Below is the link to the electronic supplementary material.

Supplementary Material 1

## Data Availability

All datasets generated or analyzed during this study are included in this published article [and its supplementary information files].

## Abbreviations

AFEX: Ammonia fiber explosion
AI: Artificial Intelligence
ANN: Artificial neural networks
CBP: Consolidated bioprocessing
CCU: Carbon capture and utilization
DA: Dilute acid
HMF: Hydroxymethylfurfural
ILs: Ionic liquids
LCA: Life-cycle analyses
LPMO: Lytic Polysaccharide Monooxygenases
ML: Machine learning
RCF: Reductive catalytic fractionation
SHCF: Separate hydrolysis and co-fermentation
SHF: Separate hydrolysis and fermentation
SSF: Simultaneous saccharification and fermentation
