# Supplementary material for: Recent advances in biomass deconstruction, microbial conversion, artificial intelligence, and carbon capture for sustainable bioenergy
Source: Bioresour Bioprocess. 2026 Jun 11;13(1):89. doi: 10.1186/s40643-026-01081-w (PMC13260523; doi:10.1186/s40643-026-01081-w)
Supplement: Supplementary file 1 — Supplementary Material 1 [file 40643_2026_1081_MOESM1_ESM.docx]

Supplementary File

**Supplementary tables**

| **Feedstock / Pretreatment** | **Dominant Inhibitors (typical range)** | **Primary Detoxification Module(s)** | **Representative Engineering** | **Key Conditions / Observations** | **References** |
| --- | --- | --- | --- | --- | --- |
| Corn stover (high solids /steam explosion) | Furfural, HMF, phenolic monomers, acetic acid from hemicellulose deacetylation. | In situ bioconversion by engineered oxidoreductases  Adaptive laboratory evolution and stress response engineering. | Overexpression of NAD(P)H-dependent oxidoreductases converting furans to less toxic alcohols.  Integration of efflux pump systems to lower intracellular concentration of inhibitors. | Combined metabolic engineering and ALE can significantly increase detoxification rates and maintain robust fermentation | (Jönsson, Alriksson and Nilvebrant, 2013a; Ujor and Okonkwo, 2022a; Shan *et al.*, 2023) |
| Sugarcane bagasse (alkaline or ammonia fiber explosion) | Ferulic acid, vanillin, HMF (~0.2 g L⁻¹), acetic acid (~3 g L⁻¹) | Laccase/peroxidase enzymatic pretreatment + redox shunt | Addition of fungal laccase (5–10 U mL⁻¹) before fermentation reduces total phenolics by 60–80%; co-expression of *pntAB* or *zwf* enhances NADPH regeneration in *Z. mobilis* | Improves ethanol productivity 1.4–1.8× and reduces lag phase | (Jönsson, Alriksson and Nilvebrant, 2013a; Adebule *et al.*, 2026) |
| Hardwood (organosolv or dilute-acid poplar, birch) | Syringaldehyde, vanillic acid, coniferyl alcohol, lignin-derived oligomers | Laccase or DyP peroxidase detox; Efflux and membrane engineering (hopanoid modulation) | Surface-immobilized laccase (2–5 U mL⁻¹) or DyP-type peroxidase in situ; *Z. mobilis* mutants with tuned *hpnF/shc* expression (hopanoid biosynthesis) for phenolic tolerance | 50% reduction in total phenolics; 2.3× increase in cell viability; improved membrane order (fluorescence anisotropy assays) | (Bollag, Shuttleworth and Anderson, 1988; Yi *et al.*, 2015; Brenac *et al.*, 2019a, 2019b) |
| Wheat straw / rice husk | HMF (~0.2–0.4 g L⁻¹), furfural (~1 g L⁻¹), ferulic acid, syringic acid | Combined enzyme–redox module + efflux activation | Co-expression of *ffr* + *fdh* and adaptive regulation of *marA* homologs; ALE under 0.8 g L⁻¹ furfural for 250 h yields upregulation of global stress genes | 3× increase in detoxification rate; stable performance in mixed-hydrolysate medium | (Jilani and Olson, 2023; Yao *et al.*, 2023) |
| Sugarcane bagasse / Corn stover (alkaline or AFEX pretreatment) | Phenolics (ferulic/para-coumaric acids), furans (furfural, HMF), acetic acid | In situ enzymatic detoxification (laccases, peroxidases)  Microbial metabolic engineering for efflux and stress response  Chemical detoxification when needed (e.g., overliming, activated carbon) | Laccase/peroxidase treatments remove phenolics from hydrolysates before fermentation.  Engineering NAD(P)H-dependent reductases enhances furans conversion | Enzymatic detoxification increases fermentability and ethanol yields, though sugar loss should be minimized.  Strains adapted or engineered for higher efflux and redox capacity show improved performance in mixed hydrolysates. | (Jönsson, Alriksson and Nilvebrant, 2013b; Sjulander and Kikas, 2020; Ujor and Okonkwo, 2022b; Shan *et al.*, 2023) |
| Softwood (pine, spruce) | Guaiacyl-type phenolics (guaiacol, vanillin), resin acids | Peroxygenase or P450-driven oxidation + efflux | Engineered *Agrocybe*peroxyg*enase* or bacterial P450 BM3 variants co-expressed in host; integration with MFS pumps (*acrB* homologs) | Up to 70% detox of guaiacol; improved ethanol yield by 1.5× | (Ibarra *et al.*, 2023; Fernández-Sandoval *et al.*, 2024) |

**Table S1. Feedstock-specific mapping of lignin-derived inhibitors and microbial detoxification strategies.** The table summarizes dominant fermentation inhibitors typically generated during pretreatment, along with primary enzymatic, redox, and membrane-based detoxification strategies. Representative genetic engineering targets, enzyme systems, and adaptive evolution approaches are highlighted, together with key operating conditions and observed improvements in microbial tolerance, viability, and ethanol productivity.

| **Feedstock** | **Carbohydrate** | **Content** | **Reference** |
| --- | --- | --- | --- |
| Corn Stover | Cellulose | 35.00% | (Ruan *et al.*, 2019) |
|  | Hemicellulose | 20% |  |
|  | Lignin | 12% |  |
| Wheat Stover | Cellulose | 39% | (Adewuyi, 2022) |
|  | Hemicellulose | 30% |  |
|  | Lignin | 16% |  |
| Rice Husk | Cellulose | 36% | (Isikgor and Becer, 2015a) |
|  | Hemicellulose | 29% |  |
|  | Lignin | 20% |  |
| Coconut Husk | Cellulose | 35% | (Salaenoi *et al.*, 2024) |
|  | Hemicellulose | 25% |  |
|  | Lignin | 16% |  |
| Coconut Shell | Cellulose | 33.61% | (M *et al.*, 2019) |
|  | Hemicellulose | 29.27% |  |
|  | Lignin | 36.51% |  |
| Giant Reed | Cellulose | 46.50% | (Vasmara *et al.*, 2023) |
|  | Hemicellulose | 29% |  |
|  | Lignin | 25.90% |  |
| Jatropha | Cellulose | 22.29% | (Wever, Heeres and Broekhuis, 2012) |
|  | Hemicellulose | 23.84% |  |
|  | Lignin | 47.60% |  |
| Sugarcane Bagasse | Cellulose | 45% | (Isikgor and Becer, 2015a) |
|  | Hemicellulose | 32% |  |
|  | Lignin | 25% |  |
| Soyabean Straw | Cellulose | 35% | (Martelli-Tosi *et al.*, 2016) |
|  | Hemicellulose | 17% |  |
|  | Lignin | 21% |  |
| Barley straw | Cellulose | 39.00% | (Pradhan *et al.*, 2025) |
|  | Hemicellulose | 26% |  |
|  | Lignin | 18% |  |
| Sorghum residue | Cellulose | 49.43% | (Andrade Alves *et al.*, 2019) |
|  | Hemicellulose | 19.18% |  |
|  | Lignin | 30.42% |  |
| Oat hulls | Cellulose | 35% | (Debiagi, Faria-Tischer and Mali, 2020) |
|  | Hemicellulose | 28% |  |
|  | Lignin | 15% |  |
| Sunflower husk | Cellulose | 46.40% | (Islamova *et al.*, 2023) |
|  | Hemicellulose | 31.60% |  |
|  | Lignin | 17.00% |  |
|  |  |  |  |
| Potato waste | Cellulose | 55% |  |
|  | Hemicellulose | 12% | (Soni *et al.*, 2023) |
|  | Lignin | 14% |  |
| Banana peels | Cellulose | 9.60% | (Acevedo *et al.*, 2021) |
|  | Hemicellulose | 9.40% |  |
|  | Lignin | 12% |  |
| Citrus peels | Cellulose | 13.60% | (Ververis *et al.*, 2007) |
|  | Hemicellulose | 6.10% |  |
|  | Lignin | 2% |  |
| Sugarbeet pulp | Cellulose | 50% | (Yasin, Gangan and Panchal, 2025) |
|  | Hemicellulose | 35% |  |
|  | Lignin | 25% |  |
| Almond shells | Cellulose | 38.48% | (Li *et al.*, 2018) |
|  | Hemicellulose | 28.82% |  |
|  | Lignin | 29.54% |  |
| Switchgrass | Cellulose | 40.08% | (Tóth *et al.*, 2023) |
|  | Hemicellulose | 38.41% |  |
|  | Lignin | 12.41% |  |
| Bamboo | Cellulose | 50% | (Alves *et al.*, 2010) |
|  | Hemicellulose | 20% |  |
|  | Lignin | 23% |  |
| Douglas fir | Cellulose | 44% | (Isikgor and Becer, 2015b) |
|  | Hemicellulose | 11% |  |
|  | Lignin | 27% |  |
| Grasses | Cellulose | 40% | (Isikgor and Becer, 2015b) |
|  | Hemicellulose | 50% |  |
|  | Lignin | 30% |  |
| Eucalyptus | Cellulose | 54% | (Alves *et al.*, 2010) |
|  | Hemicellulose | 18.40% |  |
|  | Lignin | 21.50% |  |
| Barley Hulls | Cellulose | 34% | (Isikgor and Becer, 2015b) |
|  | Hemicellulose | 36% |  |
|  | Lignin | 19% |  |
| Spruce | Cellulose | 45.50% | (Isikgor and Becer, 2015b) |
|  | Hemicellulose | 22.90% |  |
|  | Lignin | 27.90% |  |

**Table S2. The table summarizes the relative contents of cellulose, hemicellulose, and lignin (expressed as percentage of dry biomass).** highlighting compositional variability across feedstocks that influences pretreatment requirements, enzymatic hydrolysis efficiency, and downstream biofuel yields.

| **Feedstock** | **Bioethanol Yield (L/Ton)** | **Conversion technology** | **References** |
| --- | --- | --- | --- |
| Citrus Peels | 586 | Continuous Immobilized Fermentation | (Mahato *et al.*, 2021) |
| Sugarcane Bagasse | 329 | Fed-batch SSF | (Hemansi and Saini, 2023) |
| Potato Waste | 284 | Organosolv pretreatment, Enzymatic hydrolysis, ethanolic fermentation, and anaerobic digestion | (Soltaninejad, Jazini and Karimi, 2022) |
| Switch Grass | 276 | Liquid hot-water and low-moisture ammonium hydroxide (LMA) pretreatments, Enzymatic hydrolysis, Separate hydrolysis and fermentation (SHF) | (Dien *et al.*, 2018) |
| Corn Stover | 259 | Simultaneous saccharification and fermentation (SSF) | (Molaverdi *et al.*, 2021) |
| Barley Straw | 246 | Separate hydrolysis and co-fermentation (SHCF) | (Díaz, Moya and Castro, 2022) |
| Sorghum Residue | 213 | alkali (NaOH) pretreatment, **Enzymatic Degradation,** co-culture fermentation | (Punia and Kumar, 2025) |
| Giant Reed | 205 | **SHF** | (Viola *et al.*, 2015) |
| Wheat Straw | 185 | Simultaneous saccharification and fermentation | (Erdei et al., 2010) |
| Oat Hulls | 179 | Chemical pretreatment with biological conversion methods (enzymatic hydrolysis and fermentation | (Baibakova *et al.*, 2017) |
| Banana Peels | 173.7 | SSF | (Sarkar *et al.*, 2020) |
| Soyabean Molasses | 162.7 | Direct fermentation (submerged alcoholic fermentation using S. cerevisiae) | (Siqueira *et al.*, 2008) |
| Rice Straw | 127 | **SHF** | (Belal, 2013) |
| Sugarbeet Pulp | 103.5 | Direct fermentation of sugar-rich substrate using *S. cerevisiae* | (Panella and Kaffka, 2010) |
| Sunflower Husks | 101.4 | SHF using *S. cerevisiae* | (Havrysh *et al.*, 2023) |
| Acacia Wood | 90 | SSF using *S. cerevisiae* | (Ko *et al.*, 2012) |
| Jatrpoha | 88.5 | Sequential hydrolytic and fermentation | (Santos, Macedo and Santos, 2014) |
| Coconut Husk | 86 | Combined pretreatment and fermentation using *Z. mobilis* | Manuscript in progress |
| Eucalyptus Wood | 83 | SSF using *S. cerevisiae* | (Ko *et al.*, 2012) |
| Almond Shell | 73.5 | SHF using *S. cerevisiae* | (Kacem *et al.*, 2016) |
| Oil Palm | 66.5 | SHF | (Farid *et al.*, 2019) |

**Table S3. Reported bioethanol yields from a range of lignocellulosic and agro-industrial feedstocks.** Bioethanol yields are expressed as liters per metric ton of dry biomass (L ton⁻¹) and reflect variability arising from feedstock composition, pretreatment severity, hydrolysis efficiency, and fermentation performance reported in the literature.

Note: All biofuel yields are reported on a feedstock mass basis and were calculated by converting reported concentrations or mass yields into liters per metric ton (L ton⁻¹). Conversions were performed using standard density values (ethanol = 0.789 kg L⁻¹) and established stoichiometric relationships for sugar fermentation.

| **Feedstock** | **Butanol Yield (L/ton feedstock)** | **Conversion technology** | **Reference** |
| --- | --- | --- | --- |
| Jatropha cake | 556 | SHF + ABE fermentation using *c. beijerinckii* | (Jiang *et al.*, 2012) |
| Banana peels | 194 | SHCF using co-culture of *Saccharomyces*[*cerevisiae*](https://www.sciencedirect.com/topics/engineering/cerevisiae) and [*Pichia*](https://www.sciencedirect.com/topics/pharmacology-toxicology-and-pharmaceutical-science/pichia)*sp.* | (Mishra *et al.*, 2020) |
| Sugarcaen Molasses | [189](https://pmc.ncbi.nlm.nih.gov/articles/PMC4980751/) | Direct ABE fermentation using *Clostridium beijerinckii* | (Thanapornsin *et al.*, 2018) |
| Cassava | 185 | ABE fermentation using C. beijerinckii BA101 | (Lépiz-Aguilar *et al.*, 2013; Huang *et al.*, 2019) |
| Palm Oil | 178 | SHF + ABE fermentation Using *Clostridium acetobutylicum* | (Komonkiat and Cheirsilp, 2013) |
| Sugarcane | 175 | SHF + ABE fermentation Using Clostridium acetobutylicum GX01 | (Pang *et al.*, 2016) |
| Barley | 173 | SHF + ABE fermentation using *Clostridium beijerinckii* | (Qureshi *et al.*, 2010) |
| Fruit residue | 173 | ABE fermentation by *Clostridium acetobutylicum* DSM 792 | (Bardone *et al.*, 2016) |
| Soybean hulls | [172](https://pmc.ncbi.nlm.nih.gov/articles/PMC4980751/) | SHF + ABE fermentation | (Amaro Bittencourt *et al.*, 2021) |
| Wheat Straw | 170 | ABE using *Clostridium beijerinckii* P260 | (Qureshi, Saha and Cotta, 2007) |
| potato waste | 168 | SHF + ABE fermentation | (Abedini, Amiri and Karimi, 2020) |
| Rice Straw | [167](https://biotechnologyforbiofuels.biomedcentral.com/articles/10.1186/s13068-019-1508-6) | ABE using *clostridial* acetobutylicum | (Ranjan, Khanna and Moholkar, 2013) |
| Sorghum | [150](https://pmc.ncbi.nlm.nih.gov/articles/PMC4980751/) | Hybrid pervaporation process | (Cai *et al.*, 2013) |
| food waste | 149 | Direct fermentation using *C. sacchroperbutylacetonicum* N1-4 | (Wang *et al.*, 2023) |
| Switchgrass | 136 | Hydrothermolysis pretreatment - ABE | (Liu *et al.*, 2015) |
| Corn (starch) | 136 | ABE fermentation (batch fermentation) using Clostridium beijerinckii BA101 | (Qureshi and Blaschek, 2001) |
| *Physaria fendleri* (Yellow Top) | 131 | SHF + ABE fermentation using *Clostridium beijerinckii* | (Qureshi *et al.*, 2019) |
| Corn Stover | 98 | SHF + ABE fermentation (ionic liquid pretreatment) using *Clostridium saccharobutylicum* | (Ding *et al.*, 2016) |
| Sugarcane Bagasse | 58 | SHF + ABE fermentation (acid hydrolysis) using Clostridium beijerinckii | (Jonglertjunya *et al.*, 2014) |
| Algae | [57](https://pmc.ncbi.nlm.nih.gov/articles/PMC4980751/) | SHF + ABE fermentation (enzymatic hydrolysis of microalgal biomass) | (Yang *et al.*, 2023) |

**Table S4. Reported bioethanol yields from a range of lignocellulosic and agro-industrial feedstocks.** Bioethanol yield is expressed as liters per metric ton of dry biomass (L ton⁻¹) and reflect variability arising from feedstock composition, pretreatment severity, hydrolysis efficiency, and fermentation performance reported in the literature.

Note: All biofuel yields are reported on a feedstock mass basis and were calculated by converting reported concentrations or mass yields into liters per metric ton (L ton⁻¹). Conversions were performed using standard density values (ethanol = 0.81 kg L⁻¹) and established stoichiometric relationships for sugar fermentation.

| **Feedstock** | **Biohydrogen yield (kg/Ton)** | **Conversion technology** | **References** |
| --- | --- | --- | --- |
| Switch grass | 30 | Thermochemical gasification | (Sarkar *et al.*, 2014) |
| Giant reed | 18.2 | Dark fermentation | (Vasmara *et al.*, 2025) |
| Almond shell | 15.7 | Hydrothermal gasification | (Safari, Javani and Yumurtaci, 2018) |
| Potato waste | 15.67 | photo fermentation | (Das and Basak, 2025) |
| Corn stover | 12.7 | Dark–photo co-fermentation (DF–PF hybrid process) | (Zhang *et al.*, 2020) |
| Sunflower husks | 7.41 | Dark fermentation using *E. coli* | (Vanyan *et al.*, 2025) |
| Soyabean straw | 5.42 | Alkaline Pretreatment, Dark fermentation using Clostridium butyricum | (Han *et al.*, 2012) |
| Sorghum residue | 5.39 | Acidic Pretreatment, Fermentation Using *Rumincoccus albus* | (Ntaikou *et al.*, 2008) |
| Wheat stover | 4.02 | Acidic pretreatment, (SSF) Dark fermentation using mix culture | (Nasirian *et al.*, 2011) |
| Orange peels | 1.87 | Dark fermentation (ultrasound-assisted pretreatment) | (Rouabhia, Álvarez-Gallego and Fernández Güelfo, 2025) |
| Sugarbeet pulp | 1.31 | Dark fermentation (ultrasound-assisted pretreatment) | (Rouabhia, Álvarez-Gallego and Fernández Güelfo, 2025) |
| Banana peels | 0.79 | Dark fermentation | (Abd-Alla *et al.*, 2025) |
| Jatrpoha | 0.78 | Dark fermentation | (Kumar and Lin, 2014) |
| Sugarcane bagasse | 0.564 | Dark fermentation | (Fangkum and Reungsang, 2011) |
| Oil palm | 0.423 | Dark fermentation | (Atif *et al.*, 2005) |
| Rice husk | 0.255 | Dark fermentation | (Lay *et al.*, 2019) |
| Barley straw | 0.027 | sequential dark and photofermentation | (Özgür and Peksel, 2013) |

**Table S5. Comparative biohydrogen yields from diverse lignocellulosic residues and energy crops.** Biohydrogen production is reported as kilograms of hydrogen per metric ton of dry feedstock (kg ton⁻¹), reflecting differences in biomass composition, pretreatment strategy, microbial pathway, and process configuration (e.g., dark fermentation, photofermentation, or integrated systems).

Note: All biohydrogen yields are reported on a dry feedstock mass basis and were calculated by converting reported volumetric or molar hydrogen production values into kilograms per metric ton (kg ton⁻¹) of biomass. Conversions were performed using standard gas properties at standard temperature and pressure (STP), where 1 mol H₂ = 2.016 g and 1 Nm³ H₂ = 0.0899 kg.

| **Feedstock** | **Biodiesel Yield (L/ton)** | **Conversion technology** | **references** |
| --- | --- | --- | --- |
| Rapeseed/Canola | 1,125 | Base catalyzed Transesterification | (Demirbas, 2009; Gong *et al.*, 2014) |
| Palm oil | 1085 | heterogeneous catalyst Transesterification | (Anguebes-Franseschi *et al.*, 2016) |
| Sunflower | 1080 | Transesterification | (Granados *et al.*, 2007) |
| Animal fat | 1057 | Acid-catalyzed esterification followed by transesterification | (Gandure *et al.*, 2017a; Toldrá-Reig, Mora and Toldrá, 2020) |
| Castor | 1045 | Alkali-catalyzed transesterification | (Keera, El Sabagh and Taman, 2018; Zulqarnain *et al.*, 2021) |
| Waste cooking oil | 1023 | Esterification followed by transesterification | (Gandure et al., 2017; Veljković et al., 2021) |
| Neem | 1,020 | Alkali-catalyzed transesterification (ethanolysis) | (Hamadou *et al.*, 2020; Iqbal *et al.*, 2024) |
| Mustard | 434 | Co-solvent assisted alkali-catalyzed transesterification | (Fadhil, Saleh and Altamer, 2020) |
| Karanja (Pongamia) | 366 | Alkali-catalyzed transesterification | (Dwivedi, Jain and Sharma, 2011; Vijay and Chandra, 2024) |
| Jatropha | 342 | Transesterification | (Otieno *et al.*, 2022) |
| cotton seed | 220 | Transesterification | (Onukwuli *et al.*, 2017; Venkatesan, John and Sivamani, 2017) |
| Rice bran oil | 190 | Alkali-catalyzed transesterification | (Ju and Ramjan Vali, 2005; Bello and Oluboba, 2014) |
| Soybean | 161 | base-catalyzed transesterification | U.S. Department of Energy. How Much Energy Does It Take to Make a Gallon of … (DOE biodiesel yield data) |
| Corn stover | 127 | Microbial lipid production + transesterification | (Dai *et al.*, 2019; Langholtz *et al.*, 2022) |

**Table S6. Comparative biodiesel yields from oil-bearing crops, animal fats, waste oils, and selected lignocellulosic feedstocks.** Biodiesel production is expressed as liters per metric ton of feedstock (L ton⁻¹), illustrating the strong dependence of yield on lipid content, oil extractability, and feedstock type.

Note: All biodiesel yields are reported on a feedstock mass basis and were calculated by converting reported oil yields or fatty acid methyl ester (FAME) contents into liters per metric ton (L ton⁻¹) of dry biomass. Conversions were performed using standard density values for biodiesel/FAME (ρ ≈ **0.88–0.90 kg L⁻¹,** typically 0.88 kg L⁻¹ at 15–20 °C).

Table S7.

| **Section** | **Key AI/ML Applications & Examples** | **References** |
| --- | --- | --- |
| Feedstock & Pretreatment | ML models optimize pretreatment; hydrochar characterization | (Tušek *et al.*, 2024) |
| Fermentation Optimization | Predictive models reduce experimentation; biodiesel yield improved (84–98%) | (Helleckes *et al.*, 2022; Awogbemi and Kallon, 2023) |
| Strain Engineering | Hybrid/training ML models enhance metabolic pathway design | (Butean *et al.*, 2025) |
| Downstream Processing | AI enables predictive separation control (e.g., distillation) | (Butean *et al.*, 2025) |
| Integration & Supply Chain | AI optimizes supply chain, integration, reduces carbon footprint | (Chauhan *et al.*, 2025) |
| Carbon Capture & Valorization | AI accelerates material discovery, improves CCS efficiency 10–30%, optimizes CO₂ conversion | (States Department of Energy, 2024; Khalili *et al.*, 2025; Tawalbeh *et al.*, 2025) |

**Table S7. Artificial Intelligence and Machine Learning Applications Across the Biofuel Value Chain.** Summary of key AI and machine learning applications in modern biorefinery systems, highlighting representative use cases and performance metrics for feedstock, pretreatment, and fermentation optimization.
